# Near to One's Heart: The Intimate Relationship Between the Placenta and Fetal Heart

**DOI:** 10.3389/fphys.2018.00629

**Published:** 2018-06-26

**Authors:** Emily J. Camm, Kimberley J. Botting, Amanda N. Sferruzzi-Perri

**Affiliations:** Department of Physiology, Development and Neuroscience and Centre for Trophoblast Research, University of Cambridge, Cambridge, United Kingdom

**Keywords:** placenta, heart, hypoxia, altered nutrition, genetic mouse models

## Abstract

The development of the fetal heart is exquisitely controlled by a multitude of factors, ranging from humoral to mechanical forces. The gatekeeper regulating many of these factors is the placenta, an external fetal organ. As such, resistance within the placental vascular bed has a direct influence on the fetal circulation and therefore, the developing heart. In addition, the placenta serves as the interface between the mother and fetus, controlling substrate exchange and release of hormones into both circulations. The intricate relationship between the placenta and fetal heart is appreciated in instances of clinical placental pathology. Abnormal umbilical cord insertion is associated with congenital heart defects. Likewise, twin-to-twin transfusion syndrome, where monochorionic twins have unequal sharing of their placenta due to inter-twin vascular anastomoses, can result in cardiac remodeling and dysfunction in both fetuses. Moreover, epidemiological studies have suggested a link between placental phenotypic traits and increased risk of cardiovascular disease in adult life. To date, the mechanistic basis of the relationships between the placenta, fetal heart development and later risk of cardiac dysfunction have not been fully elucidated. However, studies using environmental exposures and gene manipulations in experimental animals are providing insights into the pathways involved. Likewise, surgical instrumentation of the maternal and fetal circulations in large animal species has enabled the manipulation of specific humoral and mechanical factors to investigate their roles in fetal cardiac development. This review will focus on such studies and what is known to date about the link between the placenta and heart development.

## Introduction

Since David Barker first documented the relationship between infant birth weight and adult onset disease (Barker and Osmond, 1986; Barker et al., 1989), there has been a revolutionary shift in thinking about how the early environment can impact life-long health and susceptibility to disease. The contribution of the placenta to this association and as an independent risk factor for future cardiovascular risk has more recently been identified. One of the first studies to link the placenta to cardiovascular disease was by Martyn and colleagues who identified that both the highest and lowest quintiles for placental efficiency (placenta weight as a proportion of birth weight) were associated with a greater number of deaths due to coronary heart disease in men born in the UK (Martyn et al., 1996). More recent studies of men born in Helsinki identified that a combination of maternal height, body mass index (BMI) and the shape of the placenta predict coronary heart disease in men (Eriksson et al., 2011). Specifically, short women who had a greater difference between the length and breadth of their placental surface were more likely to have a son with an increased risk of coronary heart disease. Likewise, tall women that either had a greater BMI and a placenta with a small surface area, or a lower BMI and reduced placental efficiency, conferred an elevated risk of coronary heart disease to their sons (Eriksson et al., 2011). Furthermore, having a thin placenta, or a large placenta area relative to birth weight, is associated with a greater incidence of sudden cardiac death in men and women, respectively (Barker et al., 2012). Altered placenta size and shape may be a reflection of a poor maternal environment, but may also contribute to a poor fetal environment. The placenta is the main interface between the mother and fetus, and regulates intrauterine development by supplying oxygen and nutrients required for fetal growth. There is now clear evidence that the placenta can adapt morphologically and functionally to supply signals arising from the mother, and demand signals from the fetus (Sferruzzi-Perri and Camm, 2016). The intricate relationship between the placenta and fetal heart is appreciated in instances of clinical placental pathology. Abnormal umbilical cord insertion (when the umbilical cord inserts abnormally into the fetal membranes instead of the center of the placenta), is associated with congenital heart defects (Albalawi et al., 2017). Likewise, twin-to-twin transfusion syndrome, where monochorionic twins have unequal sharing of their placenta due to inter-twin vascular anastomoses, results in one twin being under-perfused with blood and the other being over-perfused, can result in cardiac remodeling and dysfunction in both fetuses (Delabaere et al., 2016; Albalawi et al., 2017). Normally the placental circulation is considered one of low vascular resistance (Trudinger et al., 1985; Thompson and Trudinger, 1990), but in instances of poor placental development associated with fetal growth restriction, deficient remodeling of uterine spiral arteries can lead to malperfusion of the placenta and an increase in placental vascular resistance, which impair the placenta's endocrine and nutrient transport functions (For review, Chaddha et al., 2004; Burton and Jauniaux, 2018). As ~45% of the combined ventricular output from the fetal heart is directed toward the placenta, an increase in placental vascular resistance may also increase cardiac afterload, thus increasing the mechanical force that the heart beats against. To date, the mechanistic basis of the relationships between the placenta, fetal heart development and later risk of cardiac dysfunction have not been fully elucidated. However, studies using environmental exposures and gene manipulations in experimental animals are providing insights into the pathways involved.

## Animal models of altered placentation, hypoxaemia and nutrient restriction

Animal studies allow for manipulation of the placenta and the maternal and fetal environments to understand the mechanisms that underlie the placenta's influence on heart health. Of the animal models that describe both placental and heart phenotypes, there are broadly three groups that best categorize them: 1. Those that reduce oxygen and nutrient delivery to the fetus (carunclectomy, umbilico-placental embolization (UPE), single umbilical artery ligation (SUAL), maternal hyperthermia in sheep, unilateral uterine artery ligation in guinea pigs and bilateral uterine artery and vein ligation in rats; 2. Those that reduce oxygen availability for transfer to the fetus (maternal inhalation hypoxia in sheep, guinea pigs and rats); and 3. Those that alter nutrient availability for the fetus (global calorie restriction, low protein diet and high-fat/high-sugar diet). However, maternal hypoxia may result in reduced maternal food intake or alterations in the placenta's ability to deliver nutrients to the fetus. Likewise, altering the maternal diet may affect placenta development and thus decrease oxygen transfer capacity. For simplicity sake, the animal models have been divided into those that report fetal hypoxaemia (Table 1) and those that do not (Table 2). A key point highlighted by both tables is that changes to placental weight do not predict cardiac outcome as reduced, increased and unaltered placenta weight may all be associated with an altered cardiac phenotype in the offspring. It is clear that work is still required to characterize the morphometry and resource supply capacity of the placenta in animal models with cardiac phenotypes, and likewise cardiac phenotype in models with altered placentation.

**Table 1:** Animal models with fetal hypoxemia and altered placenta and heart.

**Animal model and experimental protocol**	**Placenta outcomes (late gestation)**	**Fetal blood gas, metabolite and hormone profile**	**Materno-fetal haemodynamic outcomes**	**Fetal cardiac outcomes (late gestation)**	**Postnatal cardiac outcome**	**References**
**Sheep**	**(Gestation** ~**150 d)**					
**Carunclectomy** surgical removal of the majority of endometrial caruncles prior to conception; causes IUGR	↓ number of placentomes ↓ total weight of placenta ↑ placental efficiency ↑ volume density of trophoblast in each placentome ↑ volume density of maternal capillaries in each placentome ↓ volume density of fetal and maternal connective tissue in the placenta ↑ surface area for exchange in each placentome ↑ VEGF, VEGFR2, TIE2 and ANGPT2 mRNA (angiogenesis) ↑ IGF2 mRNA (growth) ↑ LC3B mRNA (autophagy)	↓ PaO_2_ ↓ %SaO_2_ ↓ CaO_2_ ↑ %Hct ↑ PaCO_2_ ↓ or = pH ↓ glucose = lactate ↓ plasma insulin ↓ plasma IGF-1 ↑ plasma IGF-2 ↑ plasma cortisol ↓ plasma T3 and T4 ↓ plasma prolactin ↑ plasma noradrenaline and adrenaline	↓ uterine artery blood flow ↓ umbilical artery blood flow = fetal SBP = fetal DBP = fetal MAP = fetal HR ↓ fetal femoral/peripheral blood flow ↑ fetal femoral/peripheral vascular resistance	↓ absolute heart weight = heart weight relative to body weight ↓ cardiomyocyte number = Ki67+ (proliferation) = TUNEL+ (apoptosis) ↑ % mononucleated cardiomyocytes ↓ % binucleated cardiomyocytes ↓ cardiomyocyte size ↑ cardiomyocyte size relative to heart weight ↑ capillary density = blood flow to whole heart, LV, RV, and septum ↓ oxygen delivery to whole heart, LV, RV, and septum ↓ glucose delivery to whole heart, LV, RV, and septum ↑ IGF2 mRNA ↑ IGF1R mRNA and protein ↑ IGF2R mRNA	=(3 weeks) or ↓ (1 year) heart weight relative to body weight ↑ LV weight relative to body weight (LV hypertrophy) (3 weeks) = LV wall thickness (1 year) • cardiomyocyte number positively associated with birth weight (1 year) ↓ total length of capillaries (3 weeks) ↓ AT_1_R protein (3 weeks) ↑ IGF2 and IGF2R mRNA (3 weeks) ↑ Akt1 and p-Akt protein (3 weeks) ↑ p-IR and p-AS160 protein (21 days) ↑ GLUT1 protein (3 weeks) = LV TGFβ, Collagen II, Collagen III, MMP2, TIMP1-3 mRNA (3 weeks)	Robinson et al., 1979; Harding et al., 1985; Owens et al., 1987, 1989; Jones et al., 1988; Phillips et al., 1996, 2001; Simonetta et al., 1997; Edwards et al., 1999; Morrison et al., 2007; Wang et al., 2011, 2013, 2015a,b; Botting et al., 2014; Poudel et al., 2015; Zhang et al., 2016; Vranas et al., 2017
**Umbilicoplacental** **embolization** from ~110 to 130 d gestation; or ~120 d gestation to birth; causes IUGR	↓ placenta weight ↓ cross-sectional area of interdigitation between fetal and maternal tissue ↑ calcium deposition	↓ PaO_2_ ↓ %SaO_2_ ↓ CaO_2_ = Hb = pH = or ↑ PaCO_2_ ↓ plasma glucose = plasma lactate = or ↑ plasma cortisol ↑ plasma noradrenaline	=(Louey et al., 2000; Thompson et al., 2011, 2013) or↓HR Murotsuki et al., 1997 = (Louey et al., 2000; Thompson et al., 2011, 2013) or ↑ MAP (Murotsuki et al., 1997) ↑ placental vascular resistance ↑ umbilical artery resistance index ↓ umbilical blood flow	↓ heart weight (Duncan et al., 2000) = (Duncan et al., 2000; Thompson et al., 2013) or ↑ (Murotsuki et al., 1997) heart weight relative to body weight ↑ LV and RV wall thickness relative to body weight (Murotsuki et al., 1997) ↑ fibrosis and procollagen I and III mRNA in RV (Thompson et al., 2013) ↑ procollagen I mRNA in LV (Thompson et al., 2013) ↑ TGFβ mRNA and protein in LV and RV (Thompson et al., 2013) = p-/Smad2and3, Smad, p-/ERK and p-/p38 protein in LV and RV (Thompson et al., 2013) ↑ protein content and protein:DNA ratio in RV (Murotsuki et al., 1997) ↑ (LV) or = (RV) % mononucleated cardiomyocytes ↓ (LV) or = (RV) % binucleated cardiomyocytes ↑ coronary artery sensitivity to vasoconstrictors (*ex vivo*)	↓ heart weight = heart weight relative to body weight (2 months)	Trudinger et al., 1987; Murotsuki et al., 1997; Duncan et al., 2000; Louey et al., 2000; Bubb et al., 2007; Thompson et al., 2011
**Single umbilical artery ligation** ~110 d gestation; causes IUGR	↓ placenta weight	↓ PaO_2_ ↓ %SaO_2_ = (Supramaniam et al., 2006; Miller et al., 2009a; Tare et al., 2014) or ↓ pH (Oyama et al., 1992) = (Oyama et al., 1992) or ↑ (Supramaniam et al., 2006) PaCO_2_ ↑ Hb and %Hct ↓ fetal glucose uptake = glucose per weight of fetus ↑ plasma cortisol (at labor)	=fetal MAP = fetal HR ↓ umbilical blood flow	↓ heart weight	=heart weight and heart weight relative to body weight (1 day) ↑ LVDP, LV +dP/dt and LV -dP/dt (1 day) = RVDP, RV +dP/dt and RV -dP/dt (1 day) ↑ I/R infarct area (1 day) ↑ coronary eNOS, COX2, collagen II mRNA (1 day) = coronary COX1, collagen I and II and tropoelastin mRNA (1 day)	Oh et al., 1975; Oyama et al., 1992; Supramaniam et al., 2006; Miller et al., 2009a,b; Tare et al., 2014
**Maternal hyperthermia** Pregnant ewes housed at 35–40 ^O^C from ~80 to 120 d gestation; causes IUGR	↓ total weight of placenta ↓ GLUT8 mRNA and protein ↑ IGF-1 protein ↑ p-mTOR, p-ERK, and p-Akt ↓ p70S6K and p-XIAP ↑cleaved caspase ↓ telomerase activity ↓ eNOS mRNA (fetal) ↑ eNOS mRNA (maternal) ↓ Tie2 mRNA ↓ Angiopoietin 2 protein (fetal) ↑ PIGF mRNA and protein (maternal) ↓ VEGF and VEGFR1 mRNA and VEGF protein (fetal)	↓ PaO_2_ ↓ %SaO_2_ = pH = PaCO2 ↑ Hb ↑ %Hct ↓ plasma glucose ↓ plasma insulin = plasma lactate = or ↑ plasma amino acids ↑ plasma cortisol in males ↑ plasma noradrenaline ↓ umbilical uptake of O_2_, glucose, lactate and 11 amino acids per kg of fetus	↓ uterine and umbilical blood flow ↑ uterine blood flow per kg of fetus ↓ umbilical blood flow per kg of fetus ↑ umbilical artery PI and resistance = (Barry et al., 2016) or ↑ fetal MAP (Galan et al., 2005; Regnault et al., 2007) = fetal HR (Galan et al., 2005; Barry et al., 2016)	↓ absolute heart weight = heart weight relative to body weight = basal LV myocardial blood flow per gram of LV tissue = basal LV myocardial oxygen delivery, oxygen uptake, and oxygen extraction efficiency = basal LV myocardial glucose delivery and uptake ↑ insulin-stimulated LV myocardial blood flow per gram of LV tissue ↑ Insulin-stimulated LV myocardial glucose delivery and uptake ↑ GLUT4 and IRβ protein ↑ glycogen		Bell et al., 1987; Walker et al., 1990; Thureen et al., 1992; Regnault et al., 2003, 2007, 2013; Limesand et al., 2004, 2006; Galan et al., 2005; Hagen et al., 2005; Barry et al., 2006, 2016; Ziebell et al., 2007; Arroyo et al., 2009; Monson et al., 2017
**Maternal hypoxia** 10–11% O_2_ from 105 to 138 d gestation; causes IUGR	=placenta weight	NB values only available for 10 days of maternal hypoxia ↓ PaO_2_ ↓ %SaO_2_ = pH = PaCO_2_ ↑ %Hct = glucose = lactate = plasma ascorbic acid ↑ plasma urate	NB values only available for 10 days of maternal hypoxia ↑ carotid and femoral artery blood flow ↓ delivery of O_2_ through the carotid and femoral artery ↑ carotid: femoral O_2_ delivery ratio = delivery of glucose through the carotid and femoral artery = carotid:femoral glucose delivery ratio	=LVDP ↑ LVEDP ↓+dP/dt and -dP/dt		Brain et al., 2015; Allison et al., 2016
**Guinea pig**	**(Gestation** ~ **68 d)**					
**Unilateral uterine artery ligation** Performed at ~30 d gestation; causes IUGR	=(Briscoe et al., 2004) or ↓ (Lafeber et al., 1984) placenta weight	↓ CaO_2_ ↓ pH ↓ plasma glucose = plasma lactate ↓ plasma insulin		↓ heart weight (Detmer et al., 1991)	=heart weight (2 months) ↑ heart weight relative to body weight (2 months) ↑ LV collagen (2 months) ↑ myofiber width (2 months) ↓ LV lumen area (2 months) = LV wall thickness (2 months) ↑ LV wall thickness/lumen area ratio (2 months)	Jones et al., 1984; Lafeber et al., 1984; Detmer et al., 1991; Briscoe et al., 2004
**Chronic maternal hypoxia** 10.5–12% O_2_ commencing in the second half of pregnancy; causes IUGR	↑ placenta weight ↑ placental efficiency ↑ fetal capillary growth ↑ fetal capillary branching and coiling ↓ fetal capillary diameter ↓ diffusion distance + hypoxyprobe-1 in cytotrophoblasts and labyrinth ↑ VEGF mRNA ↑ PGF mRNA ↓ PAPPA, PTGS2, COMT mRNA (Preeclampsia markers)	↑ %Hct	↑ maternal MAP	↑ HIF-1a and HIF-2a mRNA and HIF-1α protein ↑ eNOS mRNA and protein in coronary arteries ↓ eNOS mRNA and protein in cardiac tissue ↑ iNOS mRNA and protein in cardiac tissue ↑ nitrite/nitrates and 3-nitrotyrosine ↑ proinflammatory cytokines ↓ apoptosis ↑ collagen ↓ cytochrome C oxidase activity	=heart weight and heart weight relative to body weight (4 months; males and females) = LV weight and LV weight relative to heart weight (4 months; males and females) ↓ (female) or = (male) cardiomyocyte number (4 months) ↓ cytochrome C oxidase activity (3 months) ↓ COX1 and COX4 protein (3 months) ↑ PPARα, FATP1, FABPpm, FATP6 and GLUT4 mRNA (4 months; males and females) ↓ (males) or = (females) FACS and AMPKα2 mRNA (4 months) = PPARβ/δ, PGC-1α, CD36, ACC, MCD, CPT-Iβ, ACADM, ACADL, ACADVL, IGF1, IGF2, IGF1R, IGF2R and ANP mRNA (4 months; males and females) ↑ (female) or = (male) p-AMPKα (4 months) = ACC, p-ACC, AS160, p-AS160, GLUT1, GLUT4, AMPKα2, p-AMPKα1, Akt1, Akt2, p-Akt (thr308), p-Akt (ser473), CaMKII, p-CamKII (thr286) and P-CaMKII (thr305) protein (4 months; males and females)	Bacon et al., 1984; Scheffen et al., 1990; Dong and Thompson, 2006; Thompson et al., 2009, 2016; Evans et al., 2012a,b; Al-Hasan et al., 2013, 2014; Botting et al., 2018
**Rat**	**(gestation** ~ **21 d)**					
**Bilateral uterine artery and vein ligation** Performed at 18 d gestation; causes IUGR*	=placental weight ↑ placental efficiency ↑ placental diameter = total placental area = labyrinth area, % of total ↑ PTHrP, PTH/PTHrP receptor and AT_1a_ mRNA ↓ PTHrP protein	=ionic and total calcium = PTHrP		=heart weight ↓ (male) or = (female) JAK2, STAT3 and GLUT1 mRNA = STAT5, PGC1α and NRF-2 mRNA ↑ (females) or = (male) SOCS3 mRNA ↓ (females) or = (male) PI3K mRNA ↑ IGF1, IGF2, Bcl2 and Cmyc mRNA (males)	↓ heart weight (1 month; males and females), but = heart weight relative to body weight ↓ heart weight (2 months; males), but = heart weight relative to body weight = heart weight and heart weight relative to body weight (6 months; females) = heart weight, but ↑ heart weight relative to body weight (6 months; males) = heart weight (13 months; postpartum females) ↓ cardiomyocyte number, but not if fostered onto a control dam (1 week; males) ↓ total cardiac protein (6 months; males) = total cardiac mRNA (6 months; males) = total and p-Akt (ser473; 6 months; males) ↑ Spp1 and Rhoa mRNA (6 months; male) ↓ Ckm mRNA (6 months; male) ↓ (male) or = (female) JAK2 mRNA (1 day, week and month) ↓ STAT3 mRNA (1 day, week and month; males) ↑ STAT3 and STAT5 mRNA (1 day; females) ↓ STAT3 mRNA (1 week; females) = STAT5 mRNA (1 day, week and month; males) ↓ (female) or ↑ (male) PGC-1α mRNA (1 week) = PGC-1α mRNA (1 week and 1 month; males and females) = NRF-2, COX III and GLUT4 mRNA (1 day, 1 week and 1 month; males and females) ↓ mtTFA mRNA (1 day males and 1 month females) ↓ MnSOD mRNA (1 month; males and females)	Wigglesworth, 1974; Wlodek et al., 2005; Wadley et al., 2010, 2013, 2016; Black et al., 2012; Cheong et al., 2016
					↑ (female) or = (male) SOD activity (1 week and 1 month) ↑ (female) or = (male) GLUT1 mRNA (1 day) = GLUT4 mRNA (1 day, 1 week and 1 month; males and females) = IGF1, IGF2, Bcl2, Cmyc mRNA (1 day; males) = IGF1, IGF2, Gata4, Nppa, Myl2 and Myh7 mRNA (1 week; males) ↑ Bcl2, Cmyc, Agtr1a and Agtr1b mRNA (1 week; males) = Nppa, Myh7, Vegfa, Col1α1, Col3, TGFβ1, Mmp2 and Timp2 (6 months; males) ↑ (females) or = (males) p-/AMPKa, p-/p38 MAPK and p-/Akt (6 months) ↑ (male) or = (female) oxidative stress (GSSG/TGSH; 6 months)	
**Maternal Hypoxia** 10.5–12% O_2_ from 15 to 21 d gestation or 6 to 21 d gestation (Zhou et al., 2013) gestation; causes IUGR	=(Phillips et al., 2017) or ↓ (Rueda-Clausen et al., 2011) placenta weight ↑ oxidative stress (DCF) ↓ trophoblast invasion (Zhou et al., 2013) • spiral artery remodeling (Zhou et al., 2013) ↑ prepro ET-1 mRNA (Zhou et al., 2013) ↑ ET_A_ and AT_1_R protein (Zhou et al., 2013) = ET_B_ and AT_2_R protein (Zhou et al., 2013)		↑ maternal SBP, DBP and MBP (Zhou et al., 2013)	=heart weight ↓ LV and septal wall thickness ↑ heart weight relative to body weight = collagen content and collagen III protein ↓ collagen I protein ↓ MMP-1 protein = MMP-2, MMP-9, TIMP1 and TIMP-2 protein ↑ MMP-13, MMP-14, TIMP-3 and TIMP-4 protein ↓ GR mRNA and protein ↑ GR promoter methylation ↓ transcription factor binding to GR exon 1 promoter ↓ CpG methylation at the CREs and Sp1 binding sites ↑ % binucleated cardiomyocytes ↓ Ki67+ ↑ apoptotic cardiomyocytes ↑ caspase 3 and 8 activity ↓ Bcl-2 protein = Bax protein ↓ Hsp70 protein ↑βAR_1_ protein = βAR_2_ protein	↓ heart weight, LV and septal wall thickness (1 week) = heart weight relative to body weight (1 week and 2 months) = (female) or ↑ (male) heart weight relative to body weight (12 months) = (female) or ↑ (male) LV weight relative to heart weight (12 months) ↑ LV cardiomyocyte size (2 months) • dilated RV and LV hypertrophy in males not females (*in vivo*; 12 months) • diastolic dysfunction (*in vivo* and *ex vivo*; 12 months) = LVDP and LVEDP (*ex vivo*; 6 months)	Bae et al., 2003; Li et al., 2003, 2004; Xu et al., 2006; Rueda-Clausen et al., 2009, 2011, 2012; Xue and Zhang, 2009; Tong et al., 2011; Zhou et al., 2013; Paradis et al., 2014; Xiong et al., 2016
					=LV contractility (*ex vivo*; 6 months) ↑ infarct size (*ex vivo*; 6 months) = (female) or ↑ (male) susceptibility to I/R injury (*ex vivo*) and MI (*in vivo*) (3 months) ↑ susceptibility to I/R injury in both males and females (12 months) = (female) or ↓ (male) PKCε and p-PKCε (3 months) = (female) or ↑ (male) lipid peroxidation (MDA; 12 months) = (female) or ↑ (male) ratio of oxidized to reduced glutathione (12 months) ↑ β/α MHC ratio (4 and 7 months) ↓ Hsp70 protein ↓ eNOS protein = cleaved caspase 3 and DNA fragmentation = βAR_1_ ↓ βAR_2_ protein ↑ collagen content and collagen I protein (1 week, 4 and 7 months) = collagen III protein (1 week) ↑ collagen III protein (4 and 7 months) ↑ MMP-1, MMP-13, TIMP-3 and TIMP-4 protein (1 week) = MMP-2, MMP-9, MMP-14, TIMP-1 and TIMP-2 protein (1 week) ↓ MMP-2 protein (4 and 7 months)	
					=plasma levels of iron homeostasis markers and myocardial iron (4 and 12 months) ↓ Ki67+ (1 week)	
13% O_2_ from 6 to 20 d gestation; does not result in IUGR	↑ placenta weight ↓ placental efficiency = placental volume, compartmental volumes and compartment to whole volume ratios ↑ Hsp70 and HNE	↑ %Hct		=heart weight, heart weight relative to body weight, LV and RV area	=heart weight and heart weight relative to body weight (4 months) = LV developed pressure and end diastolic pressure (4 months) ↑ LV contractility (4 months) ↑ responsiveness to β_1_-adrenoreceptor agonists (4 months) ↓ reactivity to muscarinic agonists (4 months) ↑ Hsp70 protein	Giussani et al., 2012; Richter et al., 2012; Kane et al., 2013

ACADL, long chain acyl-CoA dehydrogenase; ACADM, medium chain acyl-CoA dehydrogenase; ACADVL, very long chain acyl-CoA dehydrogenase; ACC, acetyl-CoA carboxylase; Agtr1a, angiotensin II receptor type 1a; Agtr1b, angiotensin II receptor type 1b; Akt, protein kinase B; AMPKα, 5′ adenosine monophosphate-activated protein kinase, α subunit; ANGPT2, angiopoietin 2; AS160, Akt substrate of 160 kilodaltons; AT_1_R, angiotensin II type 1 receptor; AT_2_R, angiotensin II type 2 receptor; β-AR, beta-adrenergic receptor; Bak, Bcl-2 homologous antagonist/killer; Bax, Bcl-2-associated X protein; Bcl2/Bcl-2, B-cell lymphoma 2 (gene/protein); CaO_2_, arterial oxygen content; CD36, fatty acid translocase; Ckm, creatine kinase muscle; Col1α1, collagen type I alpha 1; Col3, collagen type III; COMT, catechol-o-methyl-transferase; COX, cytochrome oxidase; CPT-Iβ, carnitine palmitonyl transferase I beta; CRE, cyclic adenosine monophosphate response element; d, day; DBP, diastolic blood pressure; DCF, 2′-7′-dichlorofuorescein; +dP/dt, the rate of left ventricular pressure rise in early systole, measure of contractility; −dP/dt, the rate of left ventricular pressure fall in diastole, measure of relaxation; eNOS, endothelial nitric oxide synthase; ERK, extracellular signal-regulated kinase; ET-1, endothelin−1; ET_A_/_B_, endothelin receptor A or B; FACS, long chain fatty acyl-CoA synthetase; FABPpm, plasma membrane fatty acid binding protein; FATP, fatty acid transport protein; GLUT, glucose transporter; GR, glucocorticoid receptor; GSSG, glutathione disulphide/oxidized glutathione; Hb, hemoglobin; Hct, haematocrit; HIF, hypoxia-inducible factor; HNE, 4-Hydroxynonenal; Hsp70, 70 kilodalton heat shock protein; HR, heart rate; IGF1/IGF-1, insulin-like growth factor 1 (gene/protein); IGF1R/IGF-1R, IGF-1 receptor (gene/protein); IGF2/IGF-2, insulin-like growth factor 2 (gene/protein); IGF2R/IGF-2R, IGF 2 receptor (gene/protein); iNOS, inducible nitric oxide synthase; I/R, ischaemia/reperfusion; IR, insulin receptor; JAK2, janus activated kinase 2; LV, left ventricle; LVDP, left ventricular developed pressure; LVEDP, left ventricular end diastolic pressure; MAP, mean arterial pressure; MBP, mean blood pressure; MCD, malonyl-CoA decarboxylase; MDA, malondialdehyde; MHC, myosin heavy chain; MI, myocardial infarct; MMP, matrix metalloproteinase; MnSOD, manganese form of superoxide dismutase; mTOR, mammalian target of rapamycin; mtTFA, mitochondrial transcription factor-A; Myh7, myosin heavy chain 7; Myl2, myosin light chain 2; Nppa, natriuretic peptide type A; NRF-2, nuclear respiratory factor−2; p-, phosphorylated form of protein; p38 MAPK, p38 mitogen-activated protein kinase; p70S6K, 70 kilodalton ribosomal protein S6 kinase 1; PaCO_2_, partial pressure of arterial carbon dioxide PaO_2_, partial pressure of arterial oxygen; PAPPA, pregnancy-associate plasma protein A; PI, pulsatility index; PIGF, placental growth factor; PGC-1α, peroxisome proliferator-activated receptor-γ coactivator-1α; PPAR, peroxisome proliferator-activated receptor; PTGS2, prostaglandin synthase−2; PTHrP, parathyroid hormone-related protein; Rhoa, Ras homolog gene family member A; RV, right ventricle; RVDP, right ventricular developed pressure; SaO_2_, saturation of hemoglobin with oxygen; SBP, systolic blood pressure; SOD, superoxide dismutase; Spp1, secreted phosphoprotein 1; Sp1, specificity protein 1; STAT, signal transducer and activator of transcription protein; T3, thyroid hormone (Triiodothyronine); T4, Tie-2, angiopoietin receptor−2; Thyroid hormone (thyroxine); TGFβ, transforming growth factor beta 1; TGSH, total glutathione; TIE2, angiopoietin receptor; TIMP, tissue inhibitor of metalloproteinase; VEGF, vascular endothelial growth factor; VEGFR, vascular endothelial growth factor receptor; XIAP, X-linked inhibitor of apoptosis protein. *Direct evidence of fetal hypoxaemia has not been published in the bilateral uterine artery and vein ligation model in rats. However, as less severe models of uterine artery ligation in other species have reported evidence of fetal hypoxaemia, it is assumed bilateral uterine vessel ligation results in fetal hypoxaemia; =, unchanged.

**Table 2:** Animal models of altered nutrition with altered placenta and heart.

**Animal model and experimental protocol**	**Placenta outcomes (late gestation)**	**Fetal blood gas, metabolite and hormone profile**	**Materno-fetal haemodynamic outcomes**	**Fetal cardiac outcomes (late gestation)**	**Postnatal cardiac outcome**	**References**
**Rat** **30% UN**^∧^ from 1 d of gestation to birth; causes IUGR, ↓ birth weight	↓ placental weight ↑ AgRP mRNA ↓ NPY mRNA ↓ POMC mRNA ↓ CART mRNA ↑ apelin mRNA ↓ APJ mRNA ↑ mtDNA content ↑ Nrf1 mRNA ↑ Tfam mRNA ↑ PGC-1α mRNA ↓ mt-co1 mRNA ↓ ATP6 mRNA ↑ mt-co2 mRNA ↑ efficiency of the mitochondrial respiratory chain ↓ UCP2 mRNA ↑ Ant1 mRNA ↓ Ant2 mRNA ↓ ATP content and ATP/ADP ratio	↓ fetal plasma apelin concentrations ↓ plasma IGF-1 ↓ plasma insulin ↓ plasma IGFBP-1 and−2			↑ systolic blood pressure (between 1 and ~11–12 months) ↑ HR (~12 months) ↓ or = in heart weight (up to ~14 months) (males and/or females)	Woodall et al., 1996a,b, 1999; Vickers et al., 2000, 2002; Riviere et al., 2005; Caminos et al., 2008; Mayeur et al., 2013, 2016
**Rat** **50% UN**^∧^ from 10 d of gestation to birth; causes IUGR	↓ placental weight ↓ Lz and Jz weights ↓ Bcl2 and Bcl-XL protein ↑ Bax and Bak protein ↑ activated caspase-3 protein, mediated in part via Fas signaling pathway ↓ PPARγ protein ↑ 11β-HSD-I protein ↓ 11β-HSD-2 protein = NR3C1 protein ↓ SLC2A3, SLC38A1, and SLC38A2 protein (GLUT3, SNAT1, SNAT2) ↑ SLC38A4 (SNAT4) protein ↑ leptin protein ↓ AQP1 protein ↑ AQP8 and AQP9 protein	↑ fetal plasma osmolality and Na+ concentrations			↑ elastin and GAG in aorta (1 day of age) ↓ collagen in aorta (1 day of age) ↑ MMP-9 mRNA in aorta (1 day of age) ↓ VEGF protein expression in aorta (1 day of age) ↑ smooth muscle in aorta due to hypertrophy (1 day of age) ↑ smooth muscle α-actin in aorta (1 day of age) ↑ lumen diameter and media thickness in aorta (1 day of age) ↑ eNOS in aortas (1 day of age) ↑ 189 miRNA in aorta (1 day of age) ↓ 29c, 183, 422b miRNA in aorta (1 day of age) ↑ systolic blood pressure (2 months) ↑ elastin in aorta (2 months) ↑ collagen in aorta (2 months) ↓ GAG in aorta (2 months) ↑ MMP-9 and MMP-2 mRNA in aorta (4 months)	Desai et al., 2005; Khorram et al., 2007a,b,c, 2010; Belkacemi et al., 2009, 2011a,b,c; Jelks et al., 2009
					↑ VEGF protein expression in aorta (4 months) ↓ 200a, 129, 215 and 200b miRNA in aorta (12 months) ↑ 337 miRNA in aorta (12 months) ↓ relative heart weight (9 months) ↑ smooth muscle in aorta due to hypertrophy (2 months) (females and/or males)	
**Rat** **50% UN** from 14 d gestation to weaning; causes IUGR	=placental weight ↓ fetus/placental weight ↓ BDNF mRNA and protein ↑ TrkB-FL mRNA = TrkB-FL and TrkB-T1 protein ↓ GLUT3 protein = GLUT1, GLUT4 protein ↓ 11β-HSD-2 mRNA	=plasma glucose ↑ plasma corticosterone	↓ utero-placental blood flow ↓ maternal cardiac output		↑ plasma B (newborns) then ↓ 2 h later ↑ plasma ACtH (newborns) ↑ MAP (6 months) ↑ pulse rate (6 months) • cross-fostering prevents ↑ MAP (6 months) (males)	Ahokas et al., 1981, 1983; Lesage et al., 2001, 2002; Mayeur et al., 2010; Wattez et al., 2014
**Guinea Pig** **10–30% UN** (30% from −28 to 34 d gestation then 10% from 35 d gestation to term); causes IUGR	↓ placental diameter, weight and volume ↑ placental/body weight ↓ Lz weight and % Lz ↓ trophoblast volume ↓ maternal and fetal blood space volume ↓ total surface area (Lz) ↑ barrier thickness ↑ EPO protein in female fetuses = EPOR protein ↑ VEGF protein in male fetuses	↑ hemoglobin ↓ plasma glucose		↑ relative heart weight	↓ absolute and relative heart weight (~1 month) = absolute heart weight (~3–4 months) ↓ relative heart weight (~3–4 months) (males and females)	Sohlstrom et al., 1998; Roberts et al., 2001a,b, 2002; Elias et al., 2016, 2017; Nevin et al., 2018
**Rat** **8% protein** from 1 d gestation to weaning; causes IUGR	↑ placental volume =, ↑ or ↓ placental weight ↓ placental/body weight ↓ Lz % ↑ surface area density and total surface area of materno-fetal interface = fetal capillary surface area, diameter or length ↓ VEGF protein = VEGF receptors and expression-regulating miRNA	=plasma corticosterone			↓ absolute heart weight (1 month) ↓β-adrenergic responsiveness and attenuated adrenergic and signaling (3 months, males) ↑ basal heart rate (3 months, males) = MAP, HR (1 month, males) =, ↓ or ↑ MAP (3–4.5 months, males) = HR (3 months, males) ↑ low frequency oscillations of systolic pressure (3 months, males) ↑ cardiovascular sympathetic tone (3 months, males) ↓ cardiac mitochondrial oxidative phosphorylation (~3 months, males) ↓ cardiac enzymatic antioxidant capacity (~3 months, males)	Snoeck et al., 1990; Doherty et al., 2003; Fernandez-Twinn et al., 2003, 2006; Hoppe et al., 2007; de Brito Alves et al., 2014, 2015, 2016; Liu et al., 2014; Nascimento et al., 2014; Barros et al., 2015; Paulino-Silva and Costa-Silva, 2016
**Mouse** **9% protein** from 1 d gestation to birth; causes IUGR	↓ placental weight ↓ maternal and fetal blood vessel length in Lz • perturbation of vascular endothelial cadherin and β-catenin protein				↑ systolic blood pressure (~3 to 12 months) ↓ relative heart weight (~6 months, females) = relative heart weight (~6 months, males)	Rutland et al., 2007; Watkins et al., 2008, 2011, 2015
**Rat** **9% protein** from 1 d gestation to birth; = fetal weight	↑ placental volume =, ↑ or ↓ placental weight ↓ 11β-HSD-2 and ↑ GSase activity ↓ 11β-HSD-2 mRNA = 11β-HSD-1 mRNA ↑ Aff3, Asns, SNAT2 mRNA ↑ ATF4, p-eIF2α protein		↓ maximal and overall relaxation to VEGF in uterine arteries		=absolute heart weight (~1 month) • alteration in cardiac fatty acids composition (~1 month) ↑ systolic blood pressure (~1–6 months) ↑ relative heart weight (~5 months, females only) • altered cardiac triacylglycerol concentration contingent on post-weaning fat intake and sex (24 h and ~3 months) ↑ cardiac PPARα mRNA (24 h and ~3 months) ↓ cardiac PPARα promoter methylation (neonate and ~3 months)	Langley and Jackson, 1994; Langley-Evans et al., 1994, 1996; Langley-Evans and Jackson, 1995; Gardner et al., 1997; Langley-Evans, 1997a,b; Langley-Evans and Nwagwu, 1998; Sherman and Langley-Evans, 2000; Aihie Sayer et al., 2001; Bertram et al., 2001; Itoh et al., 2002; Jackson et al., 2002; Koumentaki et al., 2002; Brawley et al., 2003; Burdge et al., 2003
					↓ eNOS mRNA levels in thoracic aorta (~3 months) = vasoconstriction of thoracic aorta (~ 4 months) • longer maximum heart rate response following isoproterenol infusion (6 months) • prenatal diet had no effect on maximal left ventricular response (6 months) = LVDP, but longer LVDP isoproterenol response in males ↑ cardiac β2-adrenergic receptors mRNA (2 weeks, females only) • impaired recovery of LVDP after ischaemia (6 months, males only) ↓ LVDP upon early reperfusion (6 months) ↑ systolic blood pressure in F2 generation, = in F3 generation (1–2 months) (males and/or females)	Torrens et al., 2003, 2006, 2008; Musha et al., 2006; Elmes et al., 2007, 2008, 2009; Harrison and Langley-Evans, 2009; Strakovsky et al., 2010; Slater-Jefferies et al., 2011; Bai et al., 2012
**Rat** **6% protein** from 1 d gestation to birth; = or ↓ fetal weight	=placental weight = Lz weight ↓ Jz weight ↓ placental efficiency ↓ number of giant and glycogen cells in Jz, thickness of Lz • altered placental mitochondrial function ↓ IGF2 (11–17KD) protein in Lz ↓ IGFBP2 protein in Lz (males only) • altered expression of marker genes for trophoblast lineages ↑ 17β-HSD-2 mRNA in Jz ↓ 11β-HSD-2 mRNA in Lz ↓ ACE2 mRNA in Lz ↓ ACE2 protein in Lz				↑ MAP (2 and 6 months) ↑ hypotensive response to ACh (12 months) ↑ hypertensive response to PE (12 months) = absolute or relative heart weight ↑ cardiac collagen fiber content, Cx43 mRNA, cardiomyocyte size (2 months) ↓ cardiomyocyte density (2 months)	Sathishkumar et al., 2009, 2012, 2015; Gao et al., 2012a,b,c, 2013; Rebelato et al., 2013, 2016; Rossini et al., 2017
**Mouse** **High fat** (12x fat) from −28 d to weaning (fetal weight not reported)	↓ placental weight ↓ number of trophoblast cells ↑ oxidative stress- mediated endothelial cell damage				↑ systolic and diastolic blood pressure (6 and 12 months, females)	Liang et al., 2009a,b, 2010
**Mouse** **High fat/high sugar** (~5x fat, ~5x sugar) from −84 d to lactation = fetal or birth weight	placental weight ↑ lipid accumulation in Db/Jz ↑ HIF1-α protein				• accelerated cardiac growth and cardiomyocyte cell hypertrophy at 3 weeks and 2 months of age, = by 3 months ↑ absolute and relative heart weight (2 months) ↑ left ventricular free wall width (2 months) ↑ cardiac NPPB, ACTA1, MHY7:MYH6 mRNA (3 weeks), = by 3 months ↑ cardiac miR-133 mRNA (2 months) ↓ cardiac GATA-4 mRNA (2 months) • activation of AKT-ERK-mTOR pathway in cardiac tissue ↓ LVDP and dP/dtmax (3 months) ↑ LVEDP (3 months) ↑ cardiac sympathetic dominance (3 months)	Fernandez-Twinn et al., 2012, 2017; Blackmore et al., 2014
**Rat** **High fat** (4.5x) from −21d to lactation; = in fetal weight or causes IUGR	↓ placental weight Lz weight ↓ Jz weight (females only) ↑ LPL, SNAT2, GLUT1, and GLUT4 mRNA (males only) ↑IL-1β, TNFα, and CD68 mRNA (males only)				↑ systolic blood pressure (~3-~5 months)	Reynolds et al., 2014, 2015; Gray et al., 2015; Albert et al., 2017

*ACE, angiotensin converting enzyme; ACh, acetylcholine; ACTH, adrenocorticotropic hormone; ACTA; actin alpha; Aft, activating transcription factor; AgRP, agouti related peptide; Ant, adenine nucleotide translocator; AKT, protein kinase B; ANT, adenosine nucleotide translocase; APJ, apelin receptor; Asns, asparagine synthetase; ATF4, activating transcription factor-4; ATP6, mitchondrially encoded ATP Synthase 6; AQP, aquaporin; Bak, Bcl-2 homologous antagonist/killer, Bax, bcl2-like protein 4; Bcl2, B-cell lymphoma 2; Bcl-XL, B-cell lymphoma-extra large; BDNF, brain-derived neurotrophic factor; CART, cocaine and amphetamine regulated transcript; CD68, cluster of differentiation 68; CPT-1, carnitine palmitoyltransferase I; Cx43, connexin 43; d, day; Db, decidua basalis; eNOS, endothelial nitric oxide synthase; EPO, erythropoietin; EPOR, erythropoietin receptor; ERK, extracellular signal-regulated kinase; GAG, glycosaminoglycans; GLUT, glucose transporter; GSase, corticosterone-inducible glutamine synthase; HIF1-α, hypoxia-inducible factor 1 alpha subunit; HR, heart rate; 11β-HSD, 11-hydroxysteroid dehydrogenase β; 17β-HSD, 17β-hydroxysteroid dehydrogenase; IGF-1, insulin-like growth factor 1; IGFBP, insulin-like growth factor binding protein; IL-1β, interleukin 1 beta; Jz, junctional zone; LPL, lipoprotein lipase; LVDP, left ventricular developed pressure; LVEDP, left ventricular end diastolic pressure; Lz, labyrinthine zone; MAP, mean atrial pressure; miRNA, microRNA; MHY, myosin heavy chain; MMP, matrix metalloproteinase; mt, mitochondrial; mt-co, mitochondrially encoded cytochrome C oxidase; mTOR, mammalian target of rapamycin; NEAA, nonessential amino acids NEAA; Nrf, Nuclear respiratory factor; NPPB, natriuretic peptide precursor B; NPY, neuropeptide Y; NR3C1, nuclear receptor subfamily 3 group C member 1; p-eIF2α, phosphorylated eukaryotic translation initiation factor 2α; PE, phenylephrine; PGC-1α, peroxisome proliferator-activated receptor-gamma coactivator 1 alpha; POMC, pro-opiomelanocortin; PPAR, peroxisome proliferator-activated receptor; SNAT, sodium-dependent neutral amino acid transporter; Tfam, transcription factor A, mitochondrial; TNFα, tumor necrosis factor alpha; TrkB-FL, tyrosine kinase receptor B full-length; TrkB-T1, tyrosine kinase receptor truncated; UCP: uncoupling protein; VEGF, vascular endothelial growth factor; UN, undernutrition. ^∧^cross-fostered to ad libitum control dam, =, unchanged*.

Due to the different benefits and limitations that come with each animal model of human development, it is the use of a range of animal models that allows for a better understanding of the influence the placenta has on both the fetal and postnatal heart. For instance, mice, rats and guinea pigs are small animals with short gestations (weeks to months) and lifespans, which allows for a high throughput of pregnancy, postnatal and intergenerational studies. The rodent and guinea pig placentae are discoid in nature, trophoblast invade and remodel the uterine vasculature to promote blood flow and the trophoblast is directly bathed in maternal blood, which is similar to the human (Adamson et al., 2002; Mess, 2007; Mess et al., 2007; Rennie et al., 2014). The caveat to using small animal models, however, is that instrumentation to repeatedly assess fetal haemodynamics and concentrations of humoral factors across gestation is not possible. As such, determining whether alterations in postnatal cardiac structure and function were present prenatally or arose in adulthood as a result of a secondary factor such as postnatal hypertension, for instance, is difficult to determine. Furthermore, mouse and rat cardiomyocytes are immature at birth and undergo their final maturation and terminal differentiation in the weeks after birth (Li et al., 1996; Soonpaa et al., 1996). This is in contrast to humans, whose pool of cardiomyocytes begin to terminally differentiate in late gestation (Kim et al., 1992), and as such, *in utero* insults may have a more profound impact on the postnatal heart. Sheep also have benefits and limitations as an experimental animal model. Due to their size, this allows for the chronic instrumentation of the fetal circulation to assess fetal haemodynamics, concentrations of humoral factors and cardiac function. However, the caveat to this is that they have a long gestation (almost 5 months) and also take a year to reach adulthood. Further, there are a limited number of facilities in the world that allow for postnatal longitudinal studies. The sheep placenta is cotyledonary in nature, composed of many individual placentomes, which form at sites in the uterus called caruncles. There is no trophoblast invasion of maternal vessels, and an epithelial layer separates maternal blood from the trophoblast (Wooding and Burton, 2008). The temporal maturation of sheep cardiomyocytes (Burrell et al., 2003; Jonker et al., 2007b), cardiac sympathetic innervation (Lebowitz et al., 1972; Lipp and Rudolph, 1972; Tucker, 1985), and maturation of the parasympathetic nervous system (Llanos et al., 1980; Yiallourou et al., 2013), however, are better matched to humans than rodents.

## Animal models with reported fetal hypoxaemia

The most common consequence of complicated pregnancy is fetal hypoxaemia, which has been reported in human intrauterine growth restriction (IUGR) (Economides et al., 1991; Mori et al., 1993; Baschat et al., 2000). One of the more comprehensive sets of paired placenta and cardiac data in a model of altered placentation comes from the carunclectomy model in sheep. The removal of the majority of endometrial caruncles from the non-pregnant uterus results in the formation of less placentomes, and reduced total placental weight and uterine blood flow in subsequent pregnancies (Robinson et al., 1979; Jones et al., 1988). This model is one of placental insufficiency from conception, and results in fetal hypoxaemia, hypoglycaemia, hypoinsulinaemia, hypercortisolaemia, hypothyroidism [reduced thyroid hormones triiodothyronine (T3) and thyroxine (T4)], elevated catecholamines (noradrenaline and adrenaline) and reduced plasma insulin-like growth factor-1 (IGF-1; for review see Morrison, 2008). T3, cortisol and IGF-1 are important modulators of cardiomyocyte growth and maturation, which are discussed below. In this model, each placentome is modified to increase its surface area for exchange between the maternal and fetal circulations, however, the fetus is still growth restricted in late gestation (Zhang et al., 2016). The fetus has reduced umbilical blood flow (although an equivalent umbilical blood flow per kg of fetus compared to controls), and is normotensive (Owens et al., 1989; Edwards et al., 1999). One of the most notable changes in fetal heart development in the carunclectomy model is a reduction in the number of cardiomyocytes in late gestation (Botting et al., 2014). Sheep, like humans, begin the transition from proliferative cardiomyocytes to terminally differentiated cardiomyocytes in late gestation (Kim et al., 1992; Burrell et al., 2003; Jonker et al., 2007b). Consequently, changes to the endowment, but also in the function of cardiomyocytes in late gestation, may have consequences throughout life. Interestingly, despite the fetus being hypoxaemic in late gestation, the fetal heart is not hypoxic, nor does it have a greater percentage of apoptotic cardiomyocyte or a diminished percentage of cardiomyocytes in the cell cycle (Botting et al., 2014). This may be due to the adaptive increase in capillary density in the fetal heart, which may increase local oxygen supply to compensate for the placental insufficiency (Botting et al., 2014). There may also be other alterations in response to placental insufficiency that decrease oxygen demand by the fetal heart, such as an increase in anaerobic metabolism, thereby protecting it from further damage. Despite there being fewer and smaller cardiomyocytes in late gestation, each cardiomyocyte is larger relative to heart weight (Morrison et al., 2007). This may suggest an alteration in the regulation of cardiomyocyte hypertrophy in late gestation due to carunclectomy. After birth, lambs born with a low birth weight develop left ventricular hypertrophy by 3 weeks of age, and have a greater signaling through the pathological hypertrophy pathway, specifically the type 2 IGF/mannose-6-phosphate receptor (IGF-2R)/Gαq/calcium calmodulin-dependent protein kinase II (CaMKII) (Wang et al., 2011, 2015b). Furthermore, the number of cardiomyocytes in the adult heart is positively correlated to birth weight (Vranas et al., 2017). Similarly, female guinea pigs exposed to maternal hypoxia have reduced cardiomyocyte number at 4 months of age, which highlights the life-long impact a reduction in cardiomyocyte endowment *in utero* may have (Botting et al., 2018). The consequence of these placenta-mediated changes on the function of the postnatal heart is yet to be determined. Further work is required to identify the contribution of low oxygen and glucose, as well as alterations in humoral factors, on the development of heart pathology in the offspring.

By comparing the carunclectomy model to UPE, which is typically induced in the last trimester in sheep, a greater understanding of the specific role of humoral and haemodynamic factors that influence the fetal heart can be determined. Infusion of insoluble microspheres into the fetal descending aorta between the renal artery and common umbilical artery results in the blockage of vessels in the fetal portion of placentomes (cotyledons), which subsequently increases placental vascular resistance and reduces gas and nutrient exchange between the fetal and maternal circulations (Trudinger et al., 1987). Similar to the carunclectomy model, UPE results in fetal hypoxaemia and elevated plasma noradrenaline concentrations, but does not lead to a persistent elevation in fetal cortisol concentrations (Louey et al., 2000; Thompson et al., 2011) or hypoglycaemia (Thompson et al., 2011). UPE results in decreased total placenta weight and IUGR. In some studies, UPE results in an increase (Murotsuki et al., 1997) or no change (Louey et al., 2000; Thompson et al., 2011, 2013) in fetal mean arterial pressure (MAP), which may be due to the timing and severity of UPE, as well as, the degree to which placental vascular resistance is increased. Interestingly, UPE studies that report elevated fetal MAP also show an increase in relative heart weight (Murotsuki et al., 1997). This is in contrast to UPE studies that report normotensive fetuses, whose heart weights may be reduced but proportional to the reduction in fetal mass (Duncan et al., 2000; Thompson et al., 2013). The difference in fetal MAP between these UPE studies highlights that elevations in fetal MAP can promote cardiac hypertrophy (discussed below). Of note, even in the absence of an elevation in fetal MAP, UPE results in an increase in fetal cardiac fibrosis and an upregulation of collagen synthesis, likely through the transforming growth factor (TGF)-β pathway (Thompson et al., 2013). Similar to the carunclectomy model, UPE results in delayed cardiomyocyte maturation, indicated by a reduced percentage of binucleated cardiomyocytes (Bubb et al., 2007; Morrison et al., 2007). However, unlike the carunclectomy model, UPE in late gestation does not alter cardiomyocyte size but decreases cell cycle activity, indicating that UPE may instead impair the proliferation of cardiomyocytes in the fetus 20 days after embolization commences (Louey et al., 2007). The differences in the cardiac phenotype between the carunclectomy and UPE models suggest that the timing of the insult in relation to placental development and cardiomyocyte maturation may help to identify the pathogenesis of cardiac pathology. Currently, the fetal and postnatal consequences of UPE on cardiac function are unknown. However, chronic fetal hypoxaemia in late gestation in sheep due to either maternal hypoxia or SUAL results in systolic and diastolic dysfunction in the isolated fetal heart (Brain et al., 2015), and greater infarct size due to ischaemia/reperfusion in the isolated newborn heart (Tare et al., 2014), respectively. Furthermore, an increase in collagen synthesis and evidence of left ventricular (LV) hypertrophy is seen in juvenile guinea pigs exposed to unilateral umbilical artery ligation from mid-gestation (Briscoe et al., 2004). Interestingly, if the insult to the placenta and fetus is shorter, but more severe, as is the case in the rat model of bilateral uterine artery and vein ligation, the cardiac phenotype with regards to the emergence of postnatal cardiac hypertrophy is less severe (heart weight relative to body weight is equivalent to controls at 2 and 6 months of age; Wadley et al., 2016). However, caution must be taken when making direct comparisons between sheep, guinea pigs, and humans relative to rats, given rat cardiomyocytes do not mature to become binucleated until after birth (binucleation occurring from 4 to 12 days after birth; Li et al., 1996). As such, rat cardiomyocytes may have a greater capacity to repair damage caused by *in utero* insults. For example, additional evidence from the same rat model of bilateral uterine vessel ligation demonstrates that a deficit in cardiomyocyte number may be corrected if a newborn is cross fostered onto a normal (sham) mother (Black et al., 2012).

Of the various sheep models of fetal hypoxaemia, one of the most documented placental phenotypes is that resulting from maternal hyperthermia. Housing pregnant ewes at 35–40°C from ~80 to 120 days of gestation results in reduced placental weight, decreased angiogenic signaling within the fetal portion of the placenta, and increased placental apoptosis compared to controls (Regnault et al., 2003; Monson et al., 2017). The maternal hyperthermia-induced placental restriction is likely due to the reported decrease in maternal uterine blood flow compared to controls. Interestingly, uterine blood flow is similar to controls when expressed per 100 g of placental and fetal weight (Regnault et al., 2007). Fetuses whose mothers were exposed to hyperthermia in pregnancy have reduced umbilical blood flow when expressed as absolute or relative to either placental or fetal weight and have increased umbilical artery pulsatility index (PI) and resistance (Regnault et al., 2007), likely due to increased placental vascular resistance. This results in either no change (Barry et al., 2016) or an increase (Galan et al., 2005; Regnault et al., 2007) in fetal MAP. Consequently, fetuses whose mothers were exposed to hyperthermia in pregnancy are hypoxaemic, hypoglycaemic, hypoinsulinaemic, hypercortisolaemic [males], and have elevated plasma noradrenaline levels compared to controls (Walker et al., 1990; Regnault et al., 1999, 2007); a humoral profile much like the carunclectomy fetus in late gestation. The hyperthermia model also induces IUGR. To date, not much is known about the impact of placental changes induced by maternal hyperthermia on the fetal heart. However, the LV of fetuses whose mothers were exposed to hyperthermia in pregnancy have a greater insulin stimulated blood flow per gram of LV tissue and insulin stimulated glucose delivery and uptake, which relates to an increase in insulin receptor (IR) and glucose transport protein 4 (GLUT4) abundance [right ventricle (RV) not reported] (Barry et al., 2016). These adaptations may increase the chance of survival in a hypoxaemic, hypoglycaemic, and hypoinsulinaemic environment, but the consequence to the postnatal heart is currently not known.

The majority of information known about the effect of hypoxia on postnatal cardiac structure and function has been obtained from rodent studies, for which information on placental phenotype is also available. Reducing the fraction of oxygen in maternal inspired air from 21% to 10.5% from days 6 and 20 of gestation in rats and guinea pigs, respectively, results in decreased trophoblast invasion and spiral artery remodeling, coupled with an increase in maternal MAP (Zhou et al., 2013; Thompson et al., 2016). However, there are beneficial changes in placental morphology that would be expected to optimize oxygen delivery to the fetus in hypoxic dams, such as an increase in vascular density and a reduction in the barrier to oxygen diffusion (Thompson et al., 2016). Despite these adaptations, the fetal heart appears to remain hypoxic as indicated by the upregulation of hypoxia-inducible factors (HIFs), markers of nitrosative damage, and reduced mitochondrial function (Thompson et al., 2009; Evans et al., 2012a; Al-Hasan et al., 2013). This fetal cardiac phenotype could also be related to impaired coronary artery function due to altered nitric oxide (NO) availability [disturbed expression of cardiac NO synthase (NOS)] (Thompson et al., 2004, 2009; Thompson and Dong, 2005; Dong and Thompson, 2006). Maternal hypoxia (10.5%) in rats from 15 to 21 day gestation induces IUGR, decreases fetal heart weight and cardiomyocyte cell cycle activity, increases cardiomyocyte apoptosis, and prematurely promotes cardiomyocyte quiescence (binucleation) (Bae et al., 2003; Paradis et al., 2014, 2015). Furthermore, offspring of mothers exposed to hypoxia in pregnancy are more susceptible to myocardial infarction and ischaemia/reperfusion injury (Li et al., 2003; Xu et al., 2006; Xue and Zhang, 2009; Rueda-Clausen et al., 2011; Shah et al., 2017). Interestingly, the presence of IUGR is not required for maternal hypoxia in rats to programme altered cardiac phenotypes. Maternal hypoxia (13%) from 6 to 20 days of gestation does not alter fetal weight compared to normoxic controls, but results in differences in LV contractility and responsiveness to α_1_-adrenergic and muscarinic agonists at 4 months of age (Giussani et al., 2012). Additionally, maternal hypoxia results in programmed vascular dysfunction in the offspring irrespective of birth weight (Morton et al., 2010; Giussani et al., 2012; Bourque et al., 2013; Brain et al., 2015).

From the aforementioned studies, oxygen clearly plays an important role in both placental and heart development, however, more information is needed to understand the direct effect oxygen plays in these associations. Moreover, the role of changes in both oxygen and nutrient availability on the placenta and heart may be more informative in the context of complicated human pregnancies, as both substrates are often altered.

## Animal models with fetal nutrient alterations, but no reported fetal hypoxaemia

An observation from the Dutch Hunger Winter Study has been the importance of timing in the programming of adult disease (Roseboom et al., 2006). Babies exposed to the famine during late gestation were born small and remained small throughout their lives, with lower rates of obesity as adults than those born before and after the famine. Conversely, babies exposed during early gestation experienced elevated rates of obesity and cardiovascular disease in later life. The Dutch Hunger Winter therefore provided valuable insight into how dietary manipulation during specific periods of development can influence subsequent health. This concept of “critical windows” during development has been tested in several different animal experimental models. Experimental studies that have manipulated maternal calorie intake or quality during pregnancy, and show alterations in both placenta and cardiovascular morphology and function, are outlined in Table 2. Overall, these studies show that the specific effects on the placenta or fetal heart depend on the type of challenge, as well as, the duration, severity and timing relative to the formation of these two organs.

Maternal calorie restriction (10–50%) and low-protein diets (6–9%) in mice, rats and guinea pigs, typically reduce placenta weight, as well as regional weights and/or volumes of the transport labyrinthine zone (Lz) and endocrine junctional zone (Jz) (Table 2). These changes are related to reduced formation of maternal blood spaces and fetal vasculature in the exchange region (Roberts et al., 2001a,b; Rutland et al., 2007), potentially mediated through vascular endothelial growth factor (VEGF) signaling (Liu et al., 2014) and/or an increase in apoptosis in the Lz (Belkacemi et al., 2009, 2011b). In addition to the effects on placental morphology, maternal undernutrition induces mitochondrial abnormalities in the placenta (Belkacemi et al., 2011b; Mayeur et al., 2013; Rebelato et al., 2013). Mitochondria are implicated in numerous critical functions for feto-placental development, including ATP production for placental growth, production of oxidative stress and hormones, and control of apoptosis (Myatt, 2006; Wakefield et al., 2011). Mitochondrial defects may modify placental activity, and could therefore contribute to the restriction of both fetal and placental growth following calorie restriction. The expression of nutrient transporters (Lesage et al., 2002; Belkacemi et al., 2011c; Reynolds et al., 2015), growth factors (Woodall et al., 1996a; Gao et al., 2012a), appetite- and metabolism-regulating peptides (Caminos et al., 2008; Mayeur et al., 2016), angiotensin-converting enzymes (Gao et al., 2012b) are also altered by calorie and protein restriction and may contribute to suboptimal fetal growth and the associated programming of adulthood hypertension in these models. The ability of the placenta to act as a barrier to circulating maternal hormones is also affected by the maternal environment. Both calorie and protein restriction in rodents alters the placental expression of 11β-hydroxysteroid dehydrogenases type 1 and 2 (Langley-Evans et al., 1996; Bertram et al., 2001; Lesage et al., 2001; Belkacemi et al., 2011c), which activate and inactivate circulating glucocorticoids, respectively. Glucocorticoids have direct effects on the heart and vasculature (Walker, 2007). Therefore, increased fetal glucocorticoid exposure due to loss of the placenta glucocorticoid barrier will adversely affect both fetal growth and cardiovascular development before birth. Maternal low protein diets or global calorie restriction, have been shown to increase systolic blood pressure or MAP in adult offspring (Table 2). The degree to which blood pressure is elevated varies with the specific nutritional challenge and potentially the extent of remodeling of the aorta and extracellular matrix (Khorram et al., 2007a,b,c, 2010), impairment in mitochondrial oxidative phosphorylation (Nascimento et al., 2014) and changes in the expression of genes and miRNAs involved in cardiac energy metabolism (Slater-Jefferies et al., 2011). Further, adult offspring who are hypertensive may also be more vulnerable to ischaemia/reperfusion injury, as seen in the 9% protein restriction model (Elmes et al., 2008). Alterations in the reactivity of resistance arteries to vasodilators or constrictors, may also contribute to elevated blood pressure in adult offspring (Brawley et al., 2003; Torrens et al., 2003, 2006, 2008; Sathishkumar et al., 2009, 2015). A maternal low protein diet results in a reduction in heart weight and endowment of cardiomyocytes at birth (Corstius et al., 2005). However, if protein restriction continues throughout lactation, during the period of cardiomyocyte maturation in rats, cardiomyocyte endowment is similar to controls at weaning (Lim et al., 2010).

In addition to undernutrition, excess calories during pregnancy can also affect the placenta and offspring heart. Maternal high-fat or high-fat/high-sugar diets have been associated with both unchanged (Fernandez-Twinn et al., 2006, 2012; Blackmore et al., 2014; Reynolds et al., 2015) and reduced fetal and placental weights (Reynolds et al., 2014, 2015), depending on the length of exposure to the obesogenic diet. A maternal high-fat/high-sugar diet increases placental lipid deposition (Fernandez-Twinn et al., 2017), expression of HIF1α (Fernandez-Twinn et al., 2017) and pro-inflammatory mediators (Reynolds et al., 2014) and alters nutrient transport in a sex-specific manner (Reynolds et al., 2014). While inflammatory processes are essential for pregnancy progression and maintenance, dysregulation of immune function is a major contributor to pregnancy-related disorders (Denison et al., 2010). However, feeding an obesogenic diet during pregnancy has been shown to result in a reduced placental fetal capillary volume (Sferruzzi-Perri et al., 2013), which would impair fetal oxygen delivery (Kulandavelu et al., 2013), thereby contributing to the hypoxia-mediated response to maternal obesity. The increase in the expression of glucose and fatty acid transporters in only male fetuses by Reynolds et al. (2014), suggests an attempt to compensate for the diet-induced placental insufficiency. A maternal high fat or high-fat/high-sugar diet is associated with increases in systolic and diastolic blood pressure, left ventricular end diastolic pressure (LVEDP), and a decrease in left ventricular developed pressure (LVDP), in young adolescent and adult offspring. A decreased LVDP and increased LVEDP, indicative of decreased ventricular compliance and impaired relaxation, respectively, is most likely related to cardiac hypertrophy (Fernandez-Twinn et al., 2012; Blackmore et al., 2014), which have been determined in the high-fat/high-sugar murine model using molecular and stereological techniques. Further work is required to characterize the fetal origins of the cardiac abnormalities observed in adult offspring of high-fat/high-sugar fed dams.

## Genetic models

Studies performed in genetically-modified mice have started to provide novel insights into the regulation of, and relationship between, fetal heart development and placental formation (Table 3). Indeed, findings of mutant mice suggest that the formation of the fetal heart requires many of the same genes that regulate the development of the placenta (e.g., *Hand1*, Firulli et al., 1998; Riley et al., 1998). The Mouse Genome Informatics database identifies 329 genes with both placental morphology *and* cardiovascular defects (search identifies 754 mutants when using broader term, extraembryonic tissue morphology in conjunction with cardiovascular; conducted on 04 February, 2018). A selection of these genes are listed in Table 3 (e.g., *Hey1/2, Mekk3, Gab1, Hai1, Flrt2, Phd2, Cited2, Ovol1, Vcam1, Mmp14/16*). Malformations of the heart and placenta are the most commonly cited reasons for mid-gestational lethality. Heart defects also arise at around day 10 of pregnancy, when organogenesis becomes highly dependent on placental function. Previous work has largely focussed on assessing the impact of a genetic manipulation on either the formation of the placenta or the fetal heart, rather than considering an interaction between the two. In spite of several of the genes listed in Table 3 being expressed in both the fetal heart and the placental cell lineages, the temporal expression and order of developmental defects have not always been accurately determined. However, some findings in mice comparing the fetal heart and placental expression of genes with respect to the time scale of development of defects, as well as, selective gene targeting strategies, have highlighted that fetal heart defects may arise secondary to placental abnormalities and/or insufficiency.

**Table 3:** Genetically-modified mice which show placental and cardiac abnormalities*.

	**Expression in placenta and fetus/fetal heart**	**Impact of constitutive loss of expression in developing conceptus (unless stated otherwise)**				**References**
**Gene**		**Placenta**	**Fetal heart development**	**Fetal viability**	**Notes**	
**Genes in the placenta important for fetal heart development**						
*Hoxa13*	Allantoic mesenchyme and the Lz fetal vessels as well as umbilical arteries	d10.5–12.5: Defective Lz vessel formation and branching	d14.5: Reduced right and left ventricular wall thickness	d11–15.5: Lethal		Shaut et al., 2008
*Ovol2*	Highest expression by the chorion and placenta with relatively low expression in fetal heart from d8.5	d9.5: Defective chorionic and Lz vascularization	d9: Small heart, defects in the growth of myocardial and endocardial layers, resulting in the abnormal looping and chamber formation	d9.5–10.5: Lethal		Unezaki et al., 2007
*P38a*	Broadly expressed by embryo (including the heart, branchial arches, limb buds and somites) and placenta (Lz and chorionic plate)	d10.5: Defective development of Lz vasculature and exchange interface	d10.5: Reduction of the myocardial cell population, thin ventricle walls, poor myocardial trabeculation	d10.5–12.5: Lethal	Tetraploid aggregation experiment (WT placenta, null embryo): Rescued fetal heart and vascular anomalies and improved viability of nulls	Adams et al., 2000; Mudgett et al., 2000
*Erk2*	Placenta and fetal organs including heart.	d10.5: Defective Lz development and vascularization	d11.5: Thin ventricular walls	d12.5: Lethal	Tetraploid aggregation experiment (WT placenta, null embryo): Rescued fetal heart defects and lethality of nulls	Hatano et al., 2003
*Fra /Fosl1*	Expressed in Lz of placenta and several fetal tissues including heart	d9.5: Failed Lz vascularization	d9.5: Dilated pericardium and presence of erythroblasts in the heart	d10.5: Lethal	Tetraploid aggregation experiment (WT placenta, null embryo): Rescued heart defect and lethality of nulls	Schreiber et al., 2000
*Pparg*	d8.5 highly expressed by trophoblast but not embryo. From d14.5 expressed by fetal brown fat	d9.5 Defective Lz vascularization defects and disorganized structure, fewer maternal blood spaces and thickened trophoblast	d9: Premature cardiomyocyte differentiation, ventricular and septum hypoplasia, myocardial thinning and degeneration of the trabecular zone	d12.5: Lethal	Tetraploid aggregation experiment (WT placenta, null embryo): Rescued cardiac defects and delayed lethality of nulls	Barak et al., 1999
*Braf*	d11.5: Expressed at highest levels in the placenta, relative to the fetus	d10.5: Defective Lz development and vascularization. Defective Jz development	d9.5: Increased heart apoptosis and defective vascularization	d11.5: Lethal	MeoxCre Braf null (WT placenta, null embryo): Rescued lethality and growth defects of nulls	Galabova-Kovacs et al., 2006
*Junb*	Ubiquitously expressed in placenta and fetus, but particularly high in placenta	d7.5: Perturbed trophoblast invasion and hormone expression d10: failure to develop and vascularise Lz	d9.5: Enlarged pericardium	d8.5–10: Lethal	Tetraploid aggregation experiment (WT placenta, null embryo): Rescued cardiac defects and improved fetal viability of nulls	Schorpp-Kistner et al., 1999
*Senp2*	From d7.5 widely expressed by trophoblast lineages in Lz and Jz. Expression in heart only observed from d10.5	d9–10.5: Impaired syncytium formation and fetal capillary branching in Lz. Reduced Jz and particularly giant cell formation	d9–10.5: Smaller heart chambers with pericardial effusion. Myocardial wall thinning and missing of atrioventricular cushions	d11.5: Lethal	Sox2Cre nulls (WT placenta, null embryo): Rescued cardiac abnormalities and embryonic lethality of nulls	Chiu et al., 2008; Maruyama et al., 2016
*E2f7/E2f8*	Most abundantly expressed by placenta relative to fetus	Placental specific loss (using cyp19cre) d10: Defective Lz formation, fewer maternal blood spaces and impaired trophoblast invasion	Placental specific loss (using cyp19cre) d10: Fetal vascular dilation and hemorrhage	Placental specific loss (using cyp19cre) d11.5: Lethal		Ouseph et al., 2012
*Ott1/Rbm15*	Expressed widely by embryo and placenta	d9.5: Defective Lz vascularization	d18.5: Ventricular septal defect	d10.5: Lethal	Sox2Cre nulls (WT placenta, null embryo): Rescued fetal growth defects and lethality of nulls	Raffel et al., 2009
*Rb*	d12.5: High expression in Lz	d12.5: Lz defective with impaired vascularization, fewer maternal blood spaces, reduced surface area and thickened trophoblast		d13.5: Lethal	Tetraploid aggregation and Meox2Cre (WT placenta, null embryo): Rescued fetal growth defects and lethality of nulls	Wu et al., 2003; Wenzel et al., 2007
**Examples of genes important for both placental and fetal heart development**						
*Mmp14/16* double KO mice (MT-MMP1/2)		d10.5: Impaired Lz vascularization and branching morphogenesis and failed formation of syncytial layers in Lz	d10.5: Dilated vasculature and enlarged pericardium	d12.5: Lethal		Szabova et al., 2010
*Flrt2*	Endothelial cells specifically in the placental Lz and epicardial and mesenchyme.	d12.5: Defective Lz development; aberrant alignment of the endothelium	d12.5: Reduced thickness of ventricular myocardium with systemic congestion	d13.5: Lethal		Tai-Nagara et al., 2017
*Hey1/2*	Both Hey genes are highly expressed in the allantois and early cardiac precursors	d10.5: Impaired Lz vascularization	d9.5: Thin myocardium trabecular defects. Impaired aortic wall formation	d14.5: Lethal		Donovan et al., 2002; Gessler et al., 2002; Fischer et al., 2004
*Mekk3/Map3k3*		d9.5: Impaired Lz formation and defective Lz angiogenesis	d10: Retarded development of the myocardium and less trabeculation	d11: Lethal		Yang et al., 2000
*Erk5*		d9.5: Defective Lz vascularization	d9.5: Abnormal cardiac looping, excessive pericardial fluid, disorganized trabeculae and myocardial lining, reduced vascularization	d10: Lethal		Regan et al., 2002
*Gab1*	Placenta and heart	d11.5: Reduced placental size, vascular density and trophoblast proliferation	d10–11.5: Blood in pericardial cavity. d13.5 ventricular hypoplasia and dilation and the thin ventricular wall	d12.5-d17: Lethal		Itoh et al., 2000
*Hai1*	Placental Lz and fetal tissues	d8.5: Thin chorionic plate and few fetal vessels d9.5: Defective Lz trophoblast differentiation and vascularization (linked to reduced Gcm1)	d10: Enlarged pericardium and thin ventricle walls	d11.5: Lethal		Tanaka et al., 2005
*Rxra*	Ubiquitously expressed	d9.5: Reduced Lz vascularization	d13.5–16.5: Thin ventricular walls, trabeculae and septum	d12–16.5: Lethal		Kastner et al., 1994; Sapin et al., 1997; Barak et al., 1999; Wendling et al., 1999; Mascrez et al., 2009
*Zfp36l1*	d8.0: Expression greatest in the allantois with low and diffuse expression in embryo	d9: Failure of the allantoic mesoderm to invaginate into the chorionic trophoblast to form the Lz. Poor Lz angiogenesis due to reduced Vegfa expression	d9.5: Less developed trabeculae and sinusoids in the myocardial wall, thin myocardial wall	d10.5: Lethal		Stumpo et al., 2004; Bell et al., 2006
*Phd2*		d10.5: Lz defective development; thickened trophoblast, large maternal blood spaces, few fetal vessels	d11.5: Defective ventricular maturation, thin ventricles, under-developed myocardial structures and trabeculae	d13.5–14.5: Lethal		Takeda et al., 2006
*Cited2*	Expressed in embryo and highly by the placenta	d12.5: Smaller placenta, impaired Lz vascularization	d13.5: Severe heart malformations including ventricle outflow and septal defects	From d14.5: Lethal		Withington et al., 2006; Lopes Floro et al., 2011; Moreau et al., 2014
*Vcam1*	d8.5–9.5: Expressed by allantois and mycardium	d8.5: Abnormal chorioallantoic fusion and Lz vascularization	d11.5: Epicardial defects			Gurtner et al., 1995; Kwee et al., 1995

Braf, Braf transforming gene; Cited2, Cbp/P300 interacting transactivator with Glu/Asp rich carboxy-terminal domain 2; d, day of gestation; E2f7/E2f8, E2F transcription factor 7; Erk2/5, extracellular signal-regulated kinase 2/5; Flrt2, fibronectin leucine rich transmembrane protein; Fra /Fosl1, FOS like 1, AP-1 transcription factor subunit; Gab1, growth factor receptor bound protein 2-associated protein 1; Gcm1, glial cells missing homolog 1; Hai1, PP2C protein (Clade A protein phosphatases type 2C); Hey1/2, hairy/enhancer-of-split related with YRPW motif 1/2; Hoxa13, Homeobox A13; Junb, JunB proto-oncogene, AP-1 transcription factor subunit; Jz, junctional zone; Lz labyrinthine zone; Mekk3/map3k3, mitogen-activated protein kinase kinase kinase 3; Mmp, matrix metalloproteinases; Ott1/Rbm15, RNA binding motif protein 15; Ovol2, ovo like zinc finger 2; P38a, mitogen activated protein kinase p38a; Phd2, egl-9 family hypoxia inducible factor 1; Pparg, peroxisome proliferator activated receptor gamma; Rb, RB transcriptional corepressor 1; Rxra, retinoid X receptor alpha; Senp2, SUMO/sentrin specific peptidase 2; WT, wildtype. *Note list is not comprehensive.

Loss of the homeobox gene transcription factor, *Hoxa13*, results in defective vascularization and formation of the placental labyrinthine (exchange) zone (Shaut et al., 2008), lethality from days 11 of gestation (Shaut et al., 2008; Scotti and Kmita, 2012) and thinning of the fetal ventricle walls. Interestingly, *Hoxa13* is expressed in cell lineages that will form the placenta, but is absent from the fetal heart (Shaut et al., 2008). A deficiency in the zinc finger transcription factor, *Ovol2* also causes abnormalities in both placenta and fetal heart development (Unezaki et al., 2007). Although, the *Ovol2* gene is primarily expressed by the chorion and placental trophoblast and only lowly expressed by the fetal heart when cardiac abnormalities arise (Unezaki et al., 2007). Collectively, these data suggest that malformations of the fetal heart may be a consequence of defects in placental development.

The expression of members of the activator protein-1 transcription factor family (*Fra1, Junb*), nuclear hormone receptors (*Pparg*), mitogen-activated protein kinase signaling pathway (*Erk2, p38a, Braf*) and protein modification machinery (*Senp2*) in the placenta also appear to be required for fetal heart development. Loss of any of these genes leads to reduced vascularization and development of the placental labyrinthine zone (Barak et al., 1999; Schorpp-Kistner et al., 1999; Adams et al., 2000; Schreiber et al., 2000; Hatano et al., 2003; Galabova-Kovacs et al., 2006; Chiu et al., 2008; Maruyama et al., 2016). These genetic deficiencies also result in thin ventricular walls, poor myocardial trabeculation, dilated pericardium and/or increased apoptosis in the fetal heart and lethality in mid-gestation (Barak et al., 1999; Schorpp-Kistner et al., 1999; Adams et al., 2000; Schreiber et al., 2000; Hatano et al., 2003; Galabova-Kovacs et al., 2006; Maruyama et al., 2016). During development, *p38a, Pparg, Braf, Junb*, and *Senp2* are more abundantly expressed by placental rather than fetal cell lineages (Adams et al., 2000; Mudgett et al., 2000), with no difference reported for *Erk2* or *Fra1*. However, tetraploid aggregation experiments and conditional gene manipulations to generate null embryos with wildtype placentas was shown to circumvent the fetal heart abnormalities and improve embryonic viability in response to *p38a, Erk2, Fra1, Pparg, Braf, Senp2*, and *Junb* deficiency (Barak et al., 1999; Schorpp-Kistner et al., 1999; Adams et al., 2000; Schreiber et al., 2000; Hatano et al., 2003; Galabova-Kovacs et al., 2006; Maruyama et al., 2016). These observations provide strong evidence that defects in the placenta were most likely to represent the primary cause of fetal cardiac defects and lethality in these mutant mice.

During the establishment of normal circulation, myocardial development and cardiac morphogenesis depend on the patterns of blood flow returning from the yolk sac and chorioallantoic placenta (Linask et al., 2014). Therefore, placental abnormalities may disrupt cardiac and vascular development by altering the haemodynamic forces of blood returning to the heart and result in fetal demise (Linask et al., 2014). In support of this, retaining expression of RNA binding gene, *Ott1/Rbm15* or the transcriptional regulator *Rb* gene in the placenta is sufficient to rescue the lethality of null fetuses (Wu et al., 2003; Raffel et al., 2009). Furthermore, the loss of placental, but not fetal expression of the transcription factor genes, *E2f7* and *E2f8*, leads to fetal vascular dilatation, multifocal hemorrhages and lethality (Ouseph et al., 2012). However, the placenta is also thought to be responsive to blood flow forces in the fetal circulation (Linask et al., 2014). Although, very little is known about the importance of the developing fetal heart for the formation of the placenta (e.g., the consequence of cardiac-specific deficiency for placentation).

## How haemodynamic changes influence the heart

Studies in fetal sheep have investigated the specific effects of altered load on the fetal heart in normoxic and euglycaemic fetuses. Specifically, increasing left ventricular afterload by partially obstructing the ascending aorta results in a thicker LV/RV wall and smaller LV chamber volume compared to control (Fishman et al., 1978). This phenomenon of left ventricular hypertrophy in response to increased afterload is seen in adults, and is a mechanism to normalize wall stress according to the law of LaPlace. In adults who have quiescent cardiomyocytes, this increase in cardiac mass is predominantly due to an increase in cardiomyocyte hypertrophy (for review, Samuel and Swynghedauw, 2008). Initially it was proposed that the increase in fetal heart mass in response to an increase in afterload was due to an increase the number of cardiomyocytes (hyperplasia) (Fishman et al., 1978). Further investigations by Jonker and colleagues determined that cardiac growth in response to increased fetal MAP and venous pressure is biphasic, initially due to cardiomyocyte hyperplasia and elongation and subsequently due to hyperplasia, premature binucleation and hypertrophy of binucleated cardiomyocytes (Jonker et al., 2007a). This phenomenon is not isolated to the LV, with an increase in pulmonary artery pressure resulting in an increase in RV weight (Segar et al., 1997). The converse is also true- obstructing blood flowing into the LV (decreasing preload) results in a smaller heart with a reduced LV/RV weight (Jonker et al., 2007a). By decreasing fetal systolic pressure with an angiotensin-converting enzyme inhibitor, O'Tierney and colleagues determined that the fetal heart is reduced in size due to a decrease in hyperplasia and not due to alteration in cardiomyocyte size (O'Tierney et al., 2010).

## How humoral factors influence cardiomyocytes either *in vivo* or *in vitro*

Treatment of fetuses *in vivo* or isolated fetal cardiomyocytes with growth factors and hormones, whose concentrations may be altered by the placenta, allows for greater understanding of how the placenta may influence the fetal heart.

### IGF-1

IGF-1 is an important growth-promoting hormone that is produced by many tissues and functions throughout fetal and postnatal development in an autocrine/paracrine fashion. IGF-1 primarily promotes growth through the type 1 IGF receptor (IGF-1R) and downstream signaling pathways, including extracellular signal-regulated kinase (ERK) and phosphoinositol-3 kinase (PI3K). The carunclectomy model in sheep (Jones et al., 1988) and undernutrition across gestation in rats (Woodall et al., 1996a), decreases fetal plasma IGF-1 concentration in late gestation. Varying results from *in vivo* experiments in fetal sheep suggest that IGF-1 can either promote cardiac growth by hypertrophy (Lumbers et al., 2009) or hyperplasia (Sundgren et al., 2003). Likewise, treating fetal sheep cardiomyocytes with a form of IGF-1 *in vitro* results in either greater (Wang et al., 2012) or equivalent (Sundgren et al., 2003) cardiomyocyte hypertrophy compared to serum-free controls. Treating neonatal rat cardiomyocytes with IGF-1 results in a similar variation of results with either cardiomyocyte hypertrophy (Bass et al., 2012) or hyperplasia (Kajstura et al., 1994) reported. Despite the inconsistency between results, IGF-1 has consistently been reported to promote fetal cardiac growth, therefore, reduced plasma concentration may in part contribute to the smaller hearts observed in fetuses from the carunclectomy and undernutrition animal models.

### Cortisol

Cortisol is an important regulatory signal during fetal development, which amongst other important roles, acts to mature the cardiovascular system in preparation for birth (for review, Fowden and Forhead, 2015). Fetuses exposed to placental insufficiency due to carunclectomy (Phillips et al., 1996) or maternal hyperthermia [males only] (Walker et al., 1990), have increased plasma cortisol concentrations compared to controls in late gestation. Cortisol infusion to fetal sheep in late gestation results in a greater heart weight accompanied by either an increase in cell cycle activity (Giraud et al., 2006; Feng et al., 2013), increased cardiomyocyte hypertrophy (Lumbers et al., 2005), or decreased DNA content in the left ventricle (Rudolph et al., 1999). Due to the inconsistency in results, it is currently unclear how an increase in cortisol may affect the fetal heart in models of placental insufficiency. However, research into the effect of other humoral factors that are regulated by cortisol, such as thyroid hormone, appear clearer.

### Thyroid hormone

Thyroid hormones, especially T3, promote the maturation of a range of organs (For review, Forhead and Fowden, 2014). T4 is produced by the fetal thyroid gland and is converted to the more active T3 in late gestation. The conversion of T4 to T3 is catalyzed by deiodinases, which are upregulated by cortisol. As such, the surge in plasma T3 concentration is concurrent with the prepartum surge in plasma cortisol concentrations. T3 infusion to fetal sheep prior to the prepartum surge in T3, results in increased cardiomyocyte binucleation [a sign of increased maturation] and decreased cardiomyocyte cell cycle activity compared to controls (Chattergoon et al., 2012a). Furthermore, surgical ablation of the fetal thyroid gland results in reduced fetal cardiomyocyte binucleation and cell cycle activity (Chattergoon et al., 2012a). *In vitro*, T3 inhibits the proliferation of cardiomyocytes isolated from hearts either before or during the prepartum surge in T3 concentration (Chattergoon et al., 2007, 2012b). The carunclectomy model in sheep results in reduced fetal T3 and T4 plasma concentrations in late gestation (Harding et al., 1985). These studies provide evidence that the decreased percentage of binucleated cardiomyocytes observed in the fetal heart from the carunclectomy (Morrison et al., 2007) and UPE (Bubb et al., 2007) models may be due to reduced plasma T3 concentrations.

## Conclusion

**Figure 1:**
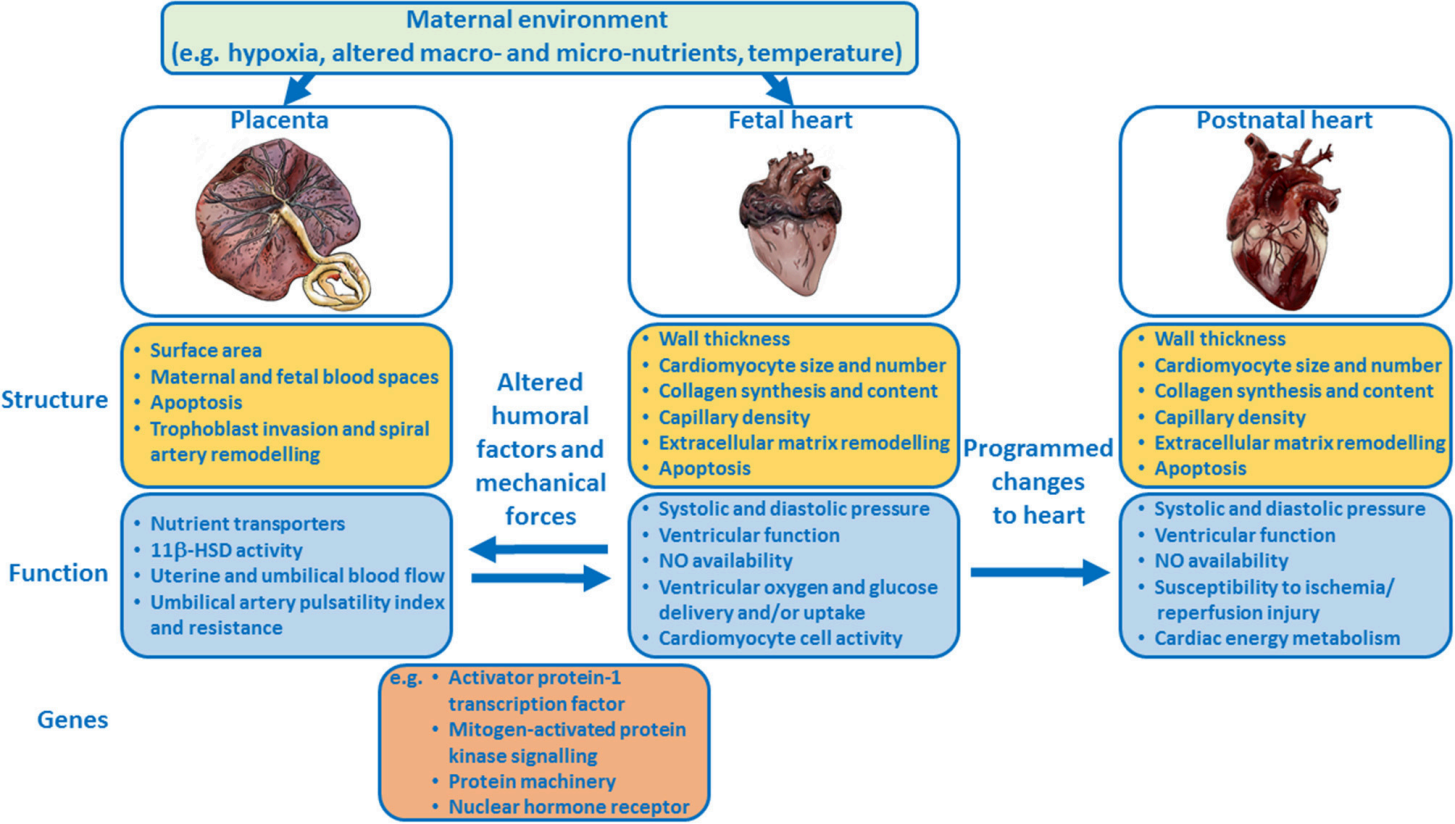
Schematic summary showing how the placenta and fetal heart may influence each other and how the maternal environment may modulate the placenta-fetal heart axis, and the structure and function of both organs. Examples of genes that regulate the development of both the placenta and heart are listed.

Epidemiological and clinical studies suggest a link between placental morphology and increased risk of cardiovascular disease in adult life. The mechanistic basis of this relationship has not been fully elucidated. However, experimental animal models and studies in genetically-modified mice, have provided novel insights into the relationship between placental formation and fetal heart development and the role humoral and mechanical forces play in the development of both of these organs (Figure 1). Further work characterizing placental morphology (e.g., surface area, thickness) and function (e.g., umbilical blood flow, oxygen and nutrient delivery) during complicated pregnancy, alongside echocardiographic measures of fetal cardiac structure, and function, will provide valuable insights into the placenta-heart axis. Such research may aid in the early diagnosis and monitoring of complicated pregnancies thus enabling timely interventions to modify long-term cardiovascular risk.

## Author contributions

EC, KB, and AS-P contributed equally to reviewing the literature and writing and editing the manuscript.

### Conflict of interest statement

The authors declare that the research was conducted in the absence of any commercial or financial relationships that could be construed as a potential conflict of interest.

## Abbreviations

BMI: body mass index
CaMKII: calcium/calmodulin-dependent protein kinase II
E: embryonic day
ERK: extracellular signal-regulated kinase
GLUT4: glucose transport protein 4
HIF: hypoxia-inducible factor
IGF-1: insulin-like growth factor-1
IGF-1R: type 1 IGF receptor
IGF-2R: type 2 IGF receptor/mannose-6-phosphate receptor
IR: insulin receptor
IUGR: intrauterine growth restriction
Jz: junctional zone
LV: left ventricle
LVDP: left ventricular developed pressure
LVEDP: left ventricular end diastolic pressure
Lz: labyrinthine zone
MAP: mean arterial pressure
NO: nitric oxide
NOS: nitric oxide synthase
PI: pulsitility index
PI3K: phosphoinositol-3 kinase
RV: right ventricle
SUAL: single umbilical artery ligation
T3: thyroid hormone (triiodothyronine)
T4: thyroid hormone (thyroxine)
TGF-β: transforming growth factor-beta
UPE: umbilico-placental embolization
VEGF: vascular endothelial growth factor.

## References

[B1] Adams R. H. Porras A. Alonso G. Jones M. Vintersten K. Panelli S. (2000). Essential role of p38alpha MAP kinase in placental but not embryonic cardiovascular development. Mol. Cell 6, 109–116. 10.1016/S1097-2765(05)00014-6 10949032

[B2] Adamson S. L. Lu Y. Whiteley K. J. Holmyard D. Hemberger M. Pfarrer C.. (2002). Interactions between trophoblast cells and the maternal and fetal circulation in the mouse placenta. Dev. Biol. 250, 358–373. 10.1006/dbio.2002.0773 12376109

[B3] Ahokas R. A. Lahaye E. B. Anderson G. D. Lipshitz J. (1981). Effect of maternal dietary restriction on fetal growth and placental transfer of alpha-amino isobutyric acid in rats. J. Nutr. 111, 2052–2058. 10.1093/jn/111.12.2052 7310531

[B4] Ahokas R. A. Anderson G. D. Lipshitz J. (1983). Effect of dietary restriction, during the last week only or throughout gestation, on cardiac output and uteroplacental blood flow in pregnant rats. J. Nutr. 113, 1766–1776. 10.1093/jn/113.9.1766 6886824

[B5] Aihie Sayer A. Dunn R. Langley-Evans S. Cooper C. (2001). Prenatal exposure to a maternal low protein diet shortens life span in rats. Gerontology 47, 9–14. 10.1159/000052764 11244286

[B6] Albalawi A. Brancusi F. Askin F. Ehsanipoor R. Wang J. Burd I.. (2017). Placental characteristics of fetuses with congenital heart disease. J. Ultrasound Med. 36, 965–972. 10.7863/ultra.16.04023 28258617

[B7] Albert B. B. Vickers M. H. Gray C. Reynolds C. M. Segovia S. A. Derraik J. G. B.. (2017). Fish oil supplementation to rats fed high-fat diet during pregnancy prevents development of impaired insulin sensitivity in male adult offspring. Sci. Rep. 7:5595. 10.1038/s41598-017-05793-0 28717143 PMC5514114

[B8] Al-Hasan Y. M. Evans L. C. Pinkas G. A. Dabkowski E. R. Stanley W. C. Thompson L. P. (2013). Chronic hypoxia impairs cytochrome oxidase activity via oxidative stress in selected fetal Guinea pig organs. Reprod. Sci. 20, 299–307. 10.1177/1933719112453509 22923417 PMC3676261

[B9] Al-Hasan Y. M. Pinkas G. A. Thompson L. P. (2014). Prenatal Hypoxia Reduces Mitochondrial protein levels and cytochrome c oxidase activity in offspring guinea pig Hearts. Reprod. Sci. 21, 883–891. 10.1177/1933719113518981 24406790 PMC4107561

[B10] Allison B. J. Brain K. L. Niu Y. Kane A. D. Herrera E. A. Thakor A. S.. (2016). Fetal *in vivo* continuous cardiovascular function during chronic hypoxia. J. Physiol. 594, 1247–1264. 10.1113/JP271091 26926316 PMC4771786

[B11] Arroyo J. A. Brown L. D. Galan H. L. (2009). Placental mammalian target of rapamycin and related signaling pathways in an ovine model of intrauterine growth restriction. Am. J. Obstet. Gynecol. 616.e1–616.e7. 10.1016/j.ajog.2009.07.031 19800600 PMC2789863

[B12] Bacon B. J. Gilbert R. D. Kaufmann P. Smith A. D. Trevino F. T. Longo L. D. (1984). Placental anatomy and diffusing capacity in guinea pigs following long-term maternal hypoxia. Placenta 5, 475–487. 10.1016/S0143-4004(84)80002-8 6527981

[B13] Bae S. Xiao Y. Li G. Casiano C. A. Zhang L. (2003). Effect of maternal chronic hypoxic exposure during gestation on apoptosis in fetal rat heart. Am. J. Physiol. Heart Circ. Physiol. 285, H983–H990. 10.1152/ajpheart.00005.2003 12750058

[B14] Bai S. Y. Briggs D. I. Vickers M. H. (2012). Increased systolic blood pressure in rat offspring following a maternal low-protein diet is normalized by maternal dietary choline supplementation. J. Dev. Origins Adult Dis. 3, 342–349. 10.1017/S2040174412000256 25102263

[B15] Barak Y. Nelson M. C. Ong E. S. Jones Y. Z. Ruiz-Lozano P. Chien K. R.. (1999). PPAR[gamma] is required for placental, cardiac, and adipose tissue development. Mol. Cell 4, 585–595. 10.1016/S1097-2765(00)80209-9 10549290

[B16] Barker D. J. Osmond C. (1986). Infant mortality, childhood nutrition, and ischaemic heart disease in England Wales. Lancet 1, 1077–1081. 10.1016/S0140-6736(86)91340-1 2871345

[B17] Barker D. J. Winter P. D. Osmond C. Margetts B. Simmonds S. J. (1989). Weight in infancy and death from ischaemic heart disease. Lancet 2, 577–580. 10.1016/S0140-6736(89)90710-1 2570282

[B18] Barker D. J. Larsen G. Osmond C. Thornburg K. L. Kajantie E. Eriksson J. G. (2012). The placental origins of sudden cardiac death. Int. J. Epidemiol. 41, 1394–1399. 10.1093/ije/dys116 22997261

[B19] Barros M. A. De Brito Alves J. L. Nogueira V. O. Wanderley A. G. Costa-Silva J. H. (2015). Maternal low-protein diet induces changes in the cardiovascular autonomic modulation in male rat offspring. Nutr Metab. Cardiovasc. Dis. 25, 123–130. 10.1016/j.numecd.2014.07.011 25287449

[B20] Barry J. S. Davidsen M. L. Limesand S. W. Galan H. L. Friedman J. E. Regnault T. R.. (2006). Developmental changes in ovine myocardial glucose transporters and insulin signaling following hyperthermia-induced intrauterine fetal growth restriction. Exp. Biol. Med. 231, 566–575. 10.1177/153537020623100511 16636305

[B21] Barry J. S. Rozance P. J. Brown L. D. Anthony R. V. Thornburg K. L. Hay W. W.. (2016). Increased fetal myocardial sensitivity to insulin-stimulated glucose metabolism during ovine fetal growth restriction. Exp. Biol. Med. 241, 839–847. 10.1177/1535370216632621 26873920 PMC4950398

[B22] Baschat A. A. Gembruch U. Reiss I. Gortner L. Weiner C. P. Harman C. R. (2000). Relationship between arterial and venous Doppler and perinatal outcome in fetal growth restriction. Ultrasound Obstet. Gynecol. 16, 407–413. 10.1046/j.1469-0705.2000.00284.x 11169323

[B23] Bass G. T. Ryall K. A. Katikapalli A. Taylor B. E. Dang S. T. Acton S. T.. (2012). Automated image analysis identifies signaling pathways regulating distinct signatures of cardiac myocyte hypertrophy. J. Mol. Cell. Cardiol. 52, 923–930. 10.1016/j.yjmcc.2011.11.009 22142594 PMC3299901

[B24] Belkacemi L. Chen C. H. Ross M. G. Desai M. (2009). Increased placental apoptosis in maternal food restricted gestations: role of the Fas pathway. Placenta 30, 739–751. 10.1016/j.placenta.2009.06.003 19616844

[B25] Belkacemi L. Desai M. Beall M. H. Liu Q. Lin J. T. Nelson D. M.. (2011a). Early compensatory adaptations in maternal undernourished pregnancies in rats: role of the aquaporins. J. Matern. Fetal Neonatal Med. 24, 752–759. 10.3109/14767058.2010.521870 20958229

[B26] Belkacemi L. Desai M. Nelson D. M. Ross M. G. (2011b). Altered mitochondrial apoptotic pathway in placentas from undernourished rat gestations. Am. J. Physiol. Regul. Integr. Comp. Physiol. 301, R1599–R1615. 10.1152/ajpregu.00100.2011 21918224 PMC3233852

[B27] Belkacemi L. Jelks A. Chen C. H. Ross M. G. Desai M. (2011c). Altered placental development in undernourished rats: role of maternal glucocorticoids. Reprod. Biol. Endocrinol. 9:105. 10.1186/1477-7827-9-105 21806804 PMC3161938

[B28] Bell A. W. Wilkening R. B. Meschia G. (1987). Some aspects of placental function in chronically heat-stressed ewes. J. Dev. Physiol. 9, 17–29. 3559063

[B29] Bell S. E. Sanchez M. J. Spasic-Boskovic O. Santalucia T. Gambardella L. Burton G. J.. (2006). The RNA binding protein Zfp36l1 is required for normal vascularisation and post-transcriptionally regulates VEGF expression. Dev. Dyn. 235, 3144–3155. 10.1002/dvdy.20949 17013884

[B30] Bertram C. Trowern A. R. Copin N. Jackson A. A. Whorwood C. B. (2001). The maternal diet during pregnancy programs altered expression of the glucocorticoid receptor and type 2 11beta-hydroxysteroid dehydrogenase: potential molecular mechanisms underlying the programming of hypertension in utero. Endocrinology 142, 2841–2853. 10.1210/endo.142.7.8238 11416003

[B31] Black M. J. Siebel A. L. Gezmish O. Moritz K. M. Wlodek M. E. (2012). Normal lactational environment restores cardiomyocyte number after uteroplacental insufficiency: implications for the preterm neonate. Am. J. Physiol. Regul. Integr. Comp. Physiol. 302, R1101–R1110. 10.1152/ajpregu.00030.2012 22403799 PMC3362141

[B32] Blackmore H. L. Niu Y. Fernandez-Twinn D. S. Tarry-Adkins J. L. Giussani D. A. Ozanne S. E. (2014). Maternal diet-induced obesity programs cardiovascular dysfunction in adult male mouse offspring independent of current body weight. Endocrinology 155, 3970–3980. 10.1210/en.2014-1383 25051449 PMC4255219

[B33] Botting K. J. McMillen I. C. Forbes H. Nyengaard J. R. Morrison J. L. (2014). Chronic hypoxemia in late gestation decreases cardiomyocyte number but does not change expression of hypoxia-responsive genes. J. Am. Heart Assoc. 3:e000531 10.1161/JAHA.113.000531 25085511 PMC4310356

[B34] Botting K. J. Loke X. Y. Zhang S. Andersen J. B. Nyengaard J. R. Morrison J. L. (2018). IUGR decreases cardiomyocyte endowment and alters cardiac metabolism in a sex and cause of IUGR specific manner. Am. J. Physiol. Regul. Integr. Comp. Physiol. [Epub ahead of print]. 10.1152/ajpregu.00180.2017 29561647

[B35] Bourque S. L. Gragasin F. S. Quon A. L. Mansour Y. Morton J. S. Davidge S. T. (2013). Prenatal hypoxia causes long-term alterations in vascular endothelin-1 function in aged male, but not female, offspring. Hypertension 62, 753–758. 10.1161/HYPERTENSIONAHA.113.01516 23940196

[B36] Brain K. L. Allison B. J. Niu Y. Cross C. M. Itani N. Kane A. D.. (2015). Induction of controlled hypoxic pregnancy in large mammalian species. Physiol. Rep. 3:e12614. 10.14814/phy2.12614 26660546 PMC4760453

[B37] Brawley L. Itoh S. Torrens C. Barker A. Bertram C. Poston L.. (2003). Dietary protein restriction in pregnancy induces hypertension and vascular defects in rat male offspring. Pediatr. Res. 54, 83–90. 10.1203/01.PDR.0000065731.00639.02 12646717

[B38] Briscoe T. A. Rehn A. E. Dieni S. Duncan J. R. Wlodek M. E. Owens J. A.. (2004). Cardiovascular and renal disease in the adolescent guinea pig after chronic placental insufficiency. Am. J. Obstet. Gynecol. 191, 847–855. 10.1016/j.ajog.2004.01.050 15467552

[B39] Bubb K. J. Cock M. L. Black M. J. Dodic M. Boon W. M. Parkington H. C.. (2007). Intrauterine growth restriction delays cardiomyocyte maturation and alters coronary artery function in the fetal sheep. J. Physiol. 578, 871–881. 10.1113/jphysiol.2006.121160 17124269 PMC2151351

[B40] Burdge G. C. Delange E. Dubois L. Dunn R. L. Hanson M. A. Jackson A. A.. (2003). Effect of reduced maternal protein intake in pregnancy in the rat on the fatty acid composition of brain, liver, plasma, heart and lung phospholipids of the offspring after weaning. Br. J. Nutr. 90, 345–352. 10.1079/BJN2003909 12908895

[B41] Burrell J. H. Boyn A. M. Kumarasamy V. Hsieh A. Head S. I. Lumbers E. R. (2003). Growth and maturation of cardiac myocytes in fetal sheep in the second half of gestation. Anat. Rec. A Discov. Mol. Cell. Evol. Biol. 274, 952–961. 10.1002/ar.a.10110 12973719

[B42] Burton G. J. Jauniaux E. (2018). Pathophysiology of placental-derived fetal growth restriction. Am. J. Obstet. Gynecol. 218, S745–S761. 10.1016/j.ajog.2017.11.577 29422210

[B43] Caminos J. E. Bravo S. B. Gonzalez C. R. Garces M. F. Cepeda L. A. Gonzalez A. C.. (2008). Food-intake-regulating-neuropeptides are expressed and regulated through pregnancy and following food restriction in rat placenta. Reprod. Biol. Endocrinol. 6:14. 10.1186/1477-7827-6-14 18384674 PMC2386475

[B44] Chaddha V. Viero S. Huppertz B. Kingdom J. (2004). Developmental biology of the placenta and the origins of placental insufficiency. Semin. Fetal Neonatal Med. 9, 357–369. 10.1016/j.siny.2004.03.006 15691771

[B45] Chattergoon N. N. Giraud G. D. Thornburg K. L. (2007). Thyroid hormone inhibits proliferation of fetal cardiac myocytes *in vitro*. J. Endocrinol. 192, R1–8. 10.1677/JOE-06-0114 17283226

[B46] Chattergoon N. N. Giraud G. D. Louey S. Stork P. Fowden A. L. Thornburg K. L. (2012a). Thyroid hormone drives fetal cardiomyocyte maturation. FASEB J. 26, 397–408. 10.1096/fj.10-179895 21974928 PMC3250248

[B47] Chattergoon N. N. Louey S. Stork P. Giraud G. D. Thornburg K. L. (2012b). Mid-gestation ovine cardiomyocytes are vulnerable to mitotic suppression by thyroid hormone. Reprod. Sci. 19, 642–649. 10.1177/1933719111432860 22421446 PMC3439123

[B48] Cheong J. N. Cuffe J. S. Jefferies A. J. Moritz K. M. Wlodek M. E. (2016). Adrenal, metabolic and cardio-renal dysfunction develops after pregnancy in rats born small or stressed by physiological measurements during pregnancy. J. Physiol. 594, 6055–6068. 10.1113/JP272212 27291586 PMC5063931

[B49] Chiu S. Y. Asai N. Costantini F. Hsu W. (2008). SUMO-specific protease 2 is essential for modulating p53-Mdm2 in development of trophoblast stem cell niches and lineages. PLoS Biol. 6:e310. 10.1371/journal.pbio.0060310 19090619 PMC2602722

[B50] Corstius H. B. Zimanyi M. A. Maka N. Herath T. Thomas W. van der Laarse A. (2005). Effect of intrauterine growth restriction on the number of cardiomyocytes in the rat heart. Pediatr. Res. 57, 796–800. 10.1203/01.PDR.0000157726.65492.CD 15774830

[B51] de Brito Alves J. L. Nogueira V. O. de Oliveira G. B. da Silva G. S. Wanderley A. G. Leandro C. G. (2014). Short- and long-term effects of a maternal low-protein diet on ventilation, O_2_/CO_2_ chemoreception and arterial blood pressure in male rat offspring. Br. J. Nutr. 111, 606–615. 10.1017/S0007114513002833 24059468

[B52] de Brito Alves J. L. Nogueira V. O. Cavalcanti Neto M. P. Leopoldino A. M. Curti C. Colombari D. S.. (2015). Maternal protein restriction increases respiratory and sympathetic activities and sensitizes peripheral chemoreflex in male rat offspring. J. Nutr. 145, 907–914. 10.3945/jn.114.202804 25934662 PMC6619683

[B53] de Brito Alves J. L. de Oliveira J. M. Ferreira D. J. Barros M. A. Nogueira V. O. Alves D. S.. (2016). Maternal protein restriction induced-hypertension is associated to oxidative disruption at transcriptional and functional levels in the medulla oblongata. Clin. Exp. Pharmacol. Physiol. 43, 1177–1184. 10.1111/1440-1681.12667 27612187

[B54] Delabaere A. Leduc F. Reboul Q. Fuchs F. Wavrant S. Fouron J. C.. (2016). Prediction of neonatal outcome of TTTS by fetal heart and Doppler ultrasound parameters before and after laser treatment. Prenat. Diagn. 36, 1199–1205. 10.1002/pd.4956 27813120

[B55] Denison F. C. Roberts K. A. Barr S. M. Norman J. E. (2010). Obesity, pregnancy, inflammation, and vascular function. Reproduction 140, 373–385. 10.1530/REP-10-0074 20215337

[B56] Desai M. Gayle D. Babu J. Ross M. G. (2005). Permanent reduction in heart and kidney organ growth in offspring of undernourished rat dams. Am. J. Obs. Gyn. 193, 1224–1232. 10.1016/j.ajog.2005.05.041 16157142

[B57] Detmer A. Gu W. Carter A. M. (1991). The blood supply to the heart and brain in the growth retarded guinea pig fetus. J. Dev. Physiol. 15, 153–160. 1940142

[B58] Doherty C. B. Lewis R. M. Sharkey A. Burton G. J. (2003). Placental composition and surface area but not vascularization are altered by maternal protein restriction in the rat. Placenta 24, 34–38. 10.1053/plac.2002.0858 12495657

[B59] Dong Y. Thompson L. P. (2006). Differential expression of endothelial nitric oxide synthase in coronary and cardiac tissue in hypoxic fetal guinea pig hearts. J. Soc. Gynecol. Investig. 13, 483–490. 10.1016/j.jsgi.2006.06.005 16979353

[B60] Donovan J. Kordylewska A. Jan Y. N. Utset M. F. (2002). Tetralogy of fallot and other congenital heart defects in Hey2 mutant mice. Curr. Biol. 12, 1605–1610. 10.1016/S0960-9822(02)01149-1 12372254

[B61] Duncan J. R. Cock M. L. Harding R. Rees S. M. (2000). Relation between damage to the placenta and the fetal brain after late-gestation placental embolization and fetal growth restriction in sheep. Am. J. Obstet. Gynecol. 183, 1013–1022. 10.1067/mob.2000.107321 11035356

[B62] Economides D. L. Nicolaides K. H. Campbell S. (1991). Metabolic and endocrine findings in appropriate and small for gestational age fetuses. J. Perinat. Med. 19, 97–105. 10.1515/jpme.1991.19.1-2.97 1651388

[B63] Edwards L. J. Simonetta G. Owens J. A. Robinson J. S. McMillen I. C. (1999). Restriction of placental and fetal growth in sheep alters fetal blood pressure responses to angiotensin, II and captopril. J. Physiol. 515, 897–904. 10.1111/j.1469-7793.1999.897ab.x 10066914 PMC2269199

[B64] Elias A. A. Ghaly A. Matushewski B. Regnault T. R. Richardson B. S. (2016). Maternal nutrient restriction in guinea pigs as an animal model for inducing fetal growth restriction. Reprod. Sci. 23, 219–227. 10.1177/1933719115602773 26342049

[B65] Elias A. A. Maki Y. Matushewski B. Nygard K. Regnault T. R. H. Richardson B. S. (2017). Maternal nutrient restriction in guinea pigs leads to fetal growth restriction with evidence for chronic hypoxia. Pediatr. Res. 82, 141–147. 10.1038/pr.2017.92 28376077

[B66] Elmes M. J. Gardner D. S. Langley-Evans S. C. (2007). Fetal exposure to a maternal low-protein diet is associated with altered left ventricular pressure response to ischaemia-reperfusion injury. Br. J. Nutr. 98, 93–100. 10.1017/S000711450769182X 17445339

[B67] Elmes M. J. McMullen S. Gardner D. S. Langley-Evans S. C. (2008). Prenatal diet determines susceptibility to cardiac ischaemia-reperfusion injury following treatment with diethylmaleic acid and N-acetylcysteine. Life Sci. 82, 149–155. 10.1016/j.lfs.2007.10.022 18062993

[B68] Elmes M. J. Haase A. Gardner D. S. Langley-Evans S. C. (2009). Sex differences in sensitivity to beta-adrenergic agonist isoproterenol in the isolated adult rat heart following prenatal protein restriction. Br. J. Nutr. 101, 725–734. 10.1017/S0007114508025075 18590591

[B69] Eriksson J. G. Kajantie E. Thornburg K. L. Osmond C. Barker D. J. (2011). Mother's body size and placental size predict coronary heart disease in men. Eur. Heart J. 32, 2297–2303. 10.1093/eurheartj/ehr147 21632601 PMC3697804

[B70] Evans L. C. Liu H. Pinkas G. A. Thompson L. P. (2012a). Chronic hypoxia increases peroxynitrite, MMP9 expression, and collagen accumulation in fetal guinea pig hearts. Pediatr. Res. 71, 25–31. 10.1038/pr.2011.10 22289847

[B71] Evans L. C. Liu H. Thompson L. P. (2012b). Differential effect of intrauterine hypoxia on caspase 3 and DNA fragmentation in fetal guinea pig hearts and brains. Reprod. Sci. 19, 298–305. 10.1177/1933719111420883 22383778 PMC3343149

[B72] Feng X. Reini S. A. Richards E. Wood C. E. Keller-Wood M. (2013). Cortisol stimulates proliferation and apoptosis in the late gestation fetal heart: differential effects of mineralocorticoid and glucocorticoid receptors. Am. J. Physiol. Regul. Integr. Comp. Physiol. 305, R343–R350. 10.1152/ajpregu.00112.2013 23785077 PMC3833392

[B73] Fernandez-Twinn D. S. Ozanne S. E. Ekizoglou S. Doherty C. James L. Gusterson B.. (2003). The maternal endocrine environment in the low-protein model of intra-uterine growth restriction. Br. J. Nutr. 90, 815–822. 10.1079/BJN2003967 13129451

[B74] Fernandez-Twinn D. S. Ekizoglou S. Wayman A. Petry C. J. Ozanne S. E. (2006). Maternal low-protein diet programs cardiac beta-adrenergic response and signaling in 3-mo-old male offspring. Am. J. Physiol. Regul. Integr. Comp. Physiol. 291, R429–R436. 10.1152/ajpregu.00608.2005 16914429

[B75] Fernandez-Twinn D. S. Blackmore H. L. Siggens L. Giussani D. A. Cross C. M. Foo R.. (2012). The programming of cardiac hypertrophy in the offspring by maternal obesity is associated with hyperinsulinemia, AKT, ERK, and mTOR activation. Endocrinology 153, 5961–5971. 10.1210/en.2012-1508 23070543 PMC3568261

[B76] Fernandez-Twinn D. S. Gascoin G. Musial B. Carr S. Duque-Guimaraes D. Blackmore H. L.. (2017). Exercise rescues obese mothers' insulin sensitivity, placental hypoxia and male offspring insulin sensitivity. Sci. Rep. 7:44650. 10.1038/srep44650 28291256 PMC5349590

[B77] Firulli A. B. McFadden D. G. Lin Q. Srivastava D. Olson E. N. (1998). Heart and extra-embryonic mesodermal defects in mouse embryos lacking the bHLH transcription factor Hand1. Nat. Genet. 18, 266–270. 10.1038/ng0398-266 9500550

[B78] Fischer A. Schumacher N. Maier M. Sendtner M. Gessler M. (2004). The Notch target genes Hey1 and Hey2 are required for embryonic vascular development. Genes Dev. 18, 901–911. 10.1101/gad.291004 15107403 PMC395849

[B79] Fishman N. H. Hof R. B. Rudolph A. M. Heymann M. A. (1978). Models of congenital heart disease in fetal lambs. Circulation 58, 354–364. 10.1161/01.CIR.58.2.354 668085

[B80] Forhead A. J. Fowden A. L. (2014). Thyroid hormones in fetal growth and prepartum maturation. J. Endocrinol. 221, R87–R103. 10.1530/JOE-14-0025 24648121

[B81] Fowden A. L. Forhead A. J. (2015). Glucocorticoids as regulatory signals during intrauterine development. Exp. Physiol. 100, 1477–1487. 10.1113/EP085212 26040783

[B82] Galabova-Kovacs G. Matzen D. Piazzolla D. Meissl K. Plyushch T. Chen A. P.. (2006). Essential role of B-Raf in ERK activation during extraembryonic development. Proc. Natl. Acad. Sci. U.S.A. 103, 1325–1330. 10.1073/pnas.0507399103 16432225 PMC1360532

[B83] Galan H. L. Anthony R. V. Rigano S. Parker T. A. de Vrijer B. Ferrazzi E.. (2005). Fetal hypertension and abnormal Doppler velocimetry in an ovine model of intrauterine growth restriction. Am. J. Obstet. Gynecol. 192, 272–279. 10.1016/j.ajog.2004.05.088 15672036

[B84] Gao H. Sathishkumar K. R. Yallampalli U. Balakrishnan M. Li X. Wu G.. (2012a). Maternal protein restriction regulates IGF2 system in placental labyrinth. Front. Biosci. 4:1434. 10.2741/472 22201967 PMC3712633

[B85] Gao H. Yallampalli U. Yallampalli C. (2012b). Maternal protein restriction reduces expression of angiotensin I-converting enzyme 2 in rat placental labyrinth zone in late pregnancy. Biol. Reprod. 86:31. 10.1095/biolreprod.111.094607 22011389 PMC3290663

[B86] Gao H. Yallampalli U. Yallampalli C. (2012c). Gestational protein restriction reduces expression of Hsd17b2 in rat placental labyrinth. Biol. Reprod. 87:68. 10.1095/biolreprod.112.100479 22837477 PMC3464908

[B87] Gao H. Yallampalli U. Yallampalli C. (2013). Gestational protein restriction affects trophoblast differentiation. Front. Biosci. 5, 591–601. 10.2741/E641 23277015 PMC4046710

[B88] Gardner D. S. Jackson A. A. Langley-Evans S. C. (1997). Maintenance of maternal diet-induced hypertension in the rat is dependent on glucocorticoids. Hypertension 30, 1525–1530. 10.1161/01.HYP.30.6.1525 9403577

[B89] Gessler M. Knobeloch K. P. Helisch A. Amann K. Schumacher N. Rohde E.. (2002). Mouse gridlock: no aortic coarctation or deficiency, but fatal cardiac defects in Hey2 -/- mice. Curr. Biol. 12, 1601–1604. 10.1016/S0960-9822(02)01150-8 12372253

[B90] Giraud G. D. Louey S. Jonker S. Schultz J. Thornburg K. L. (2006). Cortisol stimulates cell cycle activity in the cardiomyocytes of the fetal sheep. Endocrinology 148, 3643–3649. 10.1210/en.2006-0061 16690807

[B91] Giussani D. A. Camm E. J. Niu Y. Richter H. G. Blanco C. E. Gottschalk R.. (2012). Developmental programming of cardiovascular dysfunction by prenatal hypoxia and oxidative stress. PLoS ONE 7:e31017. 10.1371/journal.pone.0031017 22348036 PMC3278440

[B92] Gray C. Harrison C. J. Segovia S. A. Reynolds C. M. Vickers M. H. (2015). Maternal salt and fat intake causes hypertension and sustained endothelial dysfunction in fetal, weanling and adult male resistance vessels. Sci. Rep. 5:9753. 10.1038/srep09753 25953742 PMC4424661

[B93] Gurtner G. C. Davis V. Li H. McCoy M. J. Sharpe A. Cybulsky M. I. (1995). Targeted disruption of the murine VCAM1 gene: essential role of VCAM-1 in chorioallantoic fusion and placentation. Genes Dev. 9, 1–14. 10.1101/gad.9.1.1 7530222

[B94] Hagen A. S. Orbus R. J. Wilkening R. B. Regnault T. R. Anthony R. V. (2005). Placental expression of angiopoietin-1, angiopoietin-2 and tie-2 during placental development in an ovine model of placental insufficiency-fetal growth restriction. Pediatr. Res. 58, 1228–1232. 10.1203/01.pdr.0000185266.23265.87 16306198

[B95] Harding J. E. Jones C. T. Robinson J. S. (1985). Studies on experimental growth retardation in sheep. The effects of a small placenta in restricting transport to and growth of the fetus. J. Dev. Physiol. 7, 427–442. 4078258

[B96] Harrison M. Langley-Evans S. C. (2009). Intergenerational programming of impaired nephrogenesis and hypertension in rats following maternal protein restriction during pregnancy. Br. J. Nutr. 101, 1020–1030. 10.1017/S0007114508057607 18778527 PMC2665257

[B97] Hatano N. Mori Y. Oh-hora M. Kosugi A. Fujikawa T. Nakai N.. (2003). Essential role for ERK2 mitogen-activated protein kinase in placental development. Genes Cells 8, 847–856. 10.1046/j.1365-2443.2003.00680.x 14622137

[B98] Hoppe C. C. Evans R. G. Moritz K. M. Cullen-McEwen L. A. Fitzgerald S. M. Dowling J.. (2007). Combined prenatal and postnatal protein restriction influences adult kidney structure, function, and arterial pressure. Am. J. Physiol. Regul. Integr. Comp. Physiol. 292, R462–R469. 10.1152/ajpregu.00079.2006 16973940

[B99] Itoh M. Yoshida Y. Nishida K. Narimatsu M. Hibi M. Hirano T. (2000). Role of Gab1 in heart, placenta, and skin development and growth factor- and cytokine-induced extracellular signal-regulated kinase mitogen-activated protein kinase activation. Mol. Cell. Biol. 20, 3695–3704. 10.1128/MCB.20.10.3695-3704.2000 10779359 PMC85666

[B100] Itoh S. Brawley L. Wheeler T. Anthony F. W. Poston L. Hanson M. A. (2002). Vasodilation to vascular endothelial growth factor in the uterine artery of the pregnant rat is blunted by low dietary protein intake. Pediatr. Res. 51, 485–491. 10.1203/00006450-200204000-00014 11919334

[B101] Jackson A. A. Dunn R. L. Marchand M. C. Langley-Evans S. C. (2002). Increased systolic blood pressure in rats induced by a maternal low-protein diet is reversed by dietary supplementation with glycine. Clin. Sci. 103, 633–639. 10.1042/cs1030633 12444916

[B102] Jelks A. Belkacemi L. Han G. Chong W. L. Ross M. G. Desai M. (2009). Paradoxical increase in maternal plasma leptin levels in food-restricted gestation: contribution by placental and adipose tissue. Reprod. Sci. 16, 665–675. 10.1177/1933719109334257 19372589

[B103] Jones C. T. Lafeber H. N. Roebuck M. M. (1984). Studies on the growth of the fetal guinea pig. Changes in plasma hormone concentration during normal and abnormal growth. J. Dev. Physiol. 6, 461–472. 6098602

[B104] Jones C. T. Gu W. Harding J. E. Price D. A. Parer J. T. (1988). Studies on the growth of the fetal sheep. Effects of surgical reduction in placental size, or experimental manipulation of uterine blood flow on plasma sulphation promoting activity and on the concentration of insulin-like growth factors I and II. J. Dev. Physiol. 10, 179–189. 3397509

[B105] Jonker S. S. Faber J. J. Anderson D. F. Thornburg K. L. Louey S. Giraud G. D. (2007a). Sequential growth of fetal sheep cardiac myocytes in response to simultaneous arterial and venous hypertension. Am. J. Physiol. Regul. Integr. Comp. Physiol. 292, R913–919. 10.1152/ajpregu.00484.2006 17023664

[B106] Jonker S. S. Zhang L. Louey S. Giraud G. D. Thornburg K. L. Faber J. J. (2007b). Myocyte enlargement, differentiation, and proliferation kinetics in the fetal sheep heart. J. Appl. Physiol. 102, 1130–1142. 10.1152/japplphysiol.00937.2006 17122375

[B107] Kajstura J. Cheng W. Reiss K. Anversa P. (1994). The IGF-1-IGF-1 receptor system modulates myocyte proliferation but not myocyte cellular hypertrophy *in vitro*. Exp. Cell Res. 215, 273–283. 10.1006/excr.1994.1343 7982470

[B108] Kane A. D. Herrera E. A. Camm E. J. Giussani D. A. (2013). Vitamin C prevents intrauterine programming of *in vivo* cardiovascular dysfunction in the rat. Circ. J. 77, 2604–2611. 10.1253/circj.CJ-13-0311 23856654

[B109] Kastner P. Grondona J. M. Mark M. Gansmuller A. LeMeur M. Decimo D.. (1994). Genetic analysis of RXR alpha developmental function: convergence of RXR and RAR signaling pathways in heart and eye morphogenesis. Cell 78, 987–1003. 10.1016/0092-8674(94)90274-7 7923367

[B110] Khorram O. Momeni M. Desai M. Ross M. G. (2007a). Nutrient restriction in utero induces remodeling of the vascular extracellular matrix in rat offspring. Reprod. Sci. 14, 73–80. 10.1177/1933719106298215 17636219

[B111] Khorram O. Momeni M. Ferrini M. Desai M. Ross M. G. (2007b). *In utero* undernutrition in rats induces increased vascular smooth muscle content in the offspring. Am. J. Obst. Gyn. 196, 486 e481–e488. 10.1016/j.ajog.2007.01.020 17466715

[B112] Khorram O. Khorram N. Momeni M. Han G. Halem J. Desai M.. (2007c). Maternal undernutrition inhibits angiogenesis in the offspring: a potential mechanism of programmed hypertension. Am. J. Physiol. Regul. Integr. Comp. Physiol. 293, R745–R753. 10.1152/ajpregu.00131.2007 17507434

[B113] Khorram O. Han G. Bagherpour R. Magee T. R. Desai M. Ross M. G.. (2010). Effect of maternal undernutrition on vascular expression of micro and messenger RNA in newborn and aging offspring. Am. J. Physiol. Regul. Integr. Comp. Physiol. 298, R1366–R1374. 10.1152/ajpregu.00704.2009 20200130 PMC2867511

[B114] Kim H. D. Kim D. J. Lee I. J. Rah B. J. Sawa Y. Schaper J. (1992). Human fetal heart development after mid-term: morphometry and ultrastructural study. J. Mol. Cell. Cardiol. 24, 949–965. 10.1016/0022-2828(92)91862-Y 1433323

[B115] Koumentaki A. Anthony F. Poston L. Wheeler T. (2002). Low-protein diet impairs vascular relaxation in virgin and pregnant rats. Clin. Sci. 102, 553–560. 10.1042/cs1020553 11980575

[B116] Kulandavelu S. Whiteley K. J. Bainbridge S. A. Qu D. Adamson S. L. (2013). Endothelial NO synthase augments fetoplacental blood flow, placental vascularization, and fetal growth in mice. Hypertension 61, 259–266. 10.1161/HYPERTENSIONAHA.112.201996 23150513

[B117] Kwee L. Baldwin H. S. Shen H. M. Stewart C. L. Buck C. Buck C. A.. (1995). Defective development of the embryonic and extraembryonic circulatory systems in vascular cell adhesion molecule (VCAM-1) deficient mice. Development 121, 489–503. 7539357 10.1242/dev.121.2.489

[B118] Lafeber H. N. Rolph T. P. Jones C. T. (1984). Studies on the growth of the fetal guinea pig. The effects of ligation of the uterine artery on organ growth and development. J. Dev. Physiol. 6, 441–459. 6526985

[B119] Langley S. C. Jackson A. A. (1994). Increased systolic blood pressure in adult rats induced by fetal exposure to maternal low protein diets. Clin. Sci. 86, 217–222. 10.1042/cs0860217 8143432

[B120] Langley-Evans S. C. Jackson A. A. (1995). Captopril normalises systolic blood pressure in rats with hypertension induced by fetal exposure to maternal low protein diets. Comp. Biochem. Physiol. 110, 223–228. 10.1016/0300-9629(94)00177-U 7712066

[B121] Langley-Evans S. C. Nwagwu M. (1998). Impaired growth and increased glucocorticoid-sensitive enzyme activities in tissues of rat fetuses exposed to maternal low protein diets. Life Sci. 63, 605–615. 10.1016/S0024-3205(98)00311-7 9718086

[B122] Langley-Evans S. C. Phillips G. J. Jackson A. A. (1994). In utero exposure to maternal low protein diets induces hypertension in weanling rats, independently of maternal blood pressure changes. Clin. Nutr. 13, 319–324. 10.1016/0261-5614(94)90056-6 16843406

[B123] Langley-Evans S. C. Phillips G. J. Benediktsson R. Gardner D. S. Edwards C. R. Jackson A. A.. (1996). Protein intake in pregnancy, placental glucocorticoid metabolism and the programming of hypertension in the rat. Placenta 17, 169–172. 10.1016/S0143-4004(96)80010-5 8730887

[B124] Langley-Evans S. C. (1997a). Hypertension induced by foetal exposure to a maternal low-protein diet, in the rat, is prevented by pharmacological blockade of maternal glucocorticoid synthesis. J. Hypertension 15, 537–544. 9170007 10.1097/00004872-199715050-00010

[B125] Langley-Evans S. C. (1997b). Maternal carbenoxolone treatment lowers birthweight and induces hypertension in the offspring of rats fed a protein-replete diet. Clin. Sci. 93, 423–429. 9486087 10.1042/cs0930423

[B126] Lebowitz E. A. Novick J. S. Rudolph A. M. (1972). Development of myocardial sympathetic innervation in the fetal lamb. Pediatr. Res. 6, 887–893. 10.1203/00006450-197212000-00006 4643537

[B127] Lesage J. Blondeau B. Grino M. Breant B. Dupouy J. P. (2001). Maternal undernutrition during late gestation induces fetal overexposure to glucocorticoids and intrauterine growth retardation, and disturbs the hypothalamo-pituitary adrenal axis in the newborn rat. Endocrinology 142, 1692–1702. 10.1210/endo.142.5.8139 11316731

[B128] Lesage J. Hahn D. Leonhardt M. Blondeau B. Breant B. Dupouy J. P. (2002). Maternal undernutrition during late gestation-induced intrauterine growth restriction in the rat is associated with impaired placental GLUT3 expression, but does not correlate with endogenous corticosterone levels. J. Endocrinol. 174, 37–43. 10.1677/joe.0.1740037 12098661

[B129] Li F. Wang X. Capasso J. M. Gerdes A. M. (1996). Rapid transition of cardiac myocytes from hyperplasia to hypertrophy during postnatal development. J. Mol. Cell. Cardiol. 28, 1737–1746 10.1006/jmcc.1996.0163 8877783

[B130] Li G. Xiao Y. Estrella J. L. Ducsay C. A. Gilbert R. D. Zhang L. (2003). Effect of fetal hypoxia on heart susceptibility to ischemia and reperfusion injury in the adult rat. J. Soc. Gynecol. Investig. 10, 265–274. 10.1016/S1071-55760300074-1 12853087

[B131] Li G. Bae S. Zhang L. (2004). Effect of prenatal hypoxia on heat stress-mediated cardioprotection in adult rat heart. Am. J. Physiol. Heart Circ. Physiol. 286, H1712–H1719 10.1152/ajpheart.00898.2003 14715507

[B132] Liang C. Oest M. E. Jones J. C. Prater M. R. (2009a). Gestational high saturated fat diet alters C57BL/6 mouse perinatal skeletal formation. Birth Defects Res. B Dev. Reprod Toxicol. 86, 362–369. 10.1002/bdrb.20204 19750487

[B133] Liang C. Oest M. E. Prater M. R. (2009b). Intrauterine exposure to high saturated fat diet elevates risk of adult-onset chronic diseases in C57BL/6 mice. Birth Defects Res. B Dev. Reprod Toxicol. 86, 377–384. 10.1002/bdrb.20206 19750488

[B134] Liang C. DeCourcy K. Prater M. R. (2010). High-saturated-fat diet induces gestational diabetes and placental vasculopathy in C57BL/6 mice. Metab. Clin. Exp. 59, 943–950. 10.1016/j.metabol.2009.10.015 20022072

[B135] Lim K. Zimanyi M. A. Black M. J. (2010). Effect of maternal protein restriction during pregnancy and lactation on the number of cardiomyocytes in the postproliferative weanling rat heart. Anat. Rec. 293, 431–437. 10.1002/ar.21084 20091884

[B136] Limesand S. W. Regnault T. R. Hay W. W. (2004). Characterization of glucose transporter 8 (GLUT8) in the ovine placenta of normal and growth restricted fetuses. Placenta 25, 70–77. 10.1016/j.placenta.2003.08.012 15013641

[B137] Limesand S. W. Rozance P. J. Zerbe G. O. Hutton J. C. Hay W. W. Jr. (2006). Attenuated insulin release and storage in fetal sheep pancreatic islets with intrauterine growth restriction. Endocrinology 147, 1488–1497. 10.1210/en.2005-0900 16339204

[B138] Linask K. K. Han M. Bravo-Valenzuela N. J. (2014). Changes in vitelline and utero-placental hemodynamics: implications for cardiovascular development. Front. Physiol. 5:390. 10.3389/fphys.2014.00390 25426076 PMC4227466

[B139] Lipp J. A. Rudolph A. M. (1972). Sympathetic nerve development in the rat and guinea-pig heart. Biol. Neonate 21, 76–82. 10.1159/000240497 4651153

[B140] Liu X. Lin Y. Tian B. Miao J. Xi C. Liu C. (2014). Maternal protein restriction alters VEGF signaling and decreases pulmonary alveolar in fetal rats. Int. J. Clin. Exp. Pathol. 7, 3101–3111. 25031729 PMC4097290

[B141] Llanos A. J. Green J. R. Creasy R. K. Rudolph A. M. (1980). Increased heart rate response to parasympathetic and beta adrenergic blockade in growth-retarded fetal lambs. Am. J. Obstet. Gynecol. 136, 808–813. 10.1016/0002-9378(80)90460-3 7355968

[B142] Lopes Floro K. Artap S. T. Preis J. I. Fatkin D. Chapman G. Furtado M. B.. (2011). Loss of Cited2 causes congenital heart disease by perturbing left-right patterning of the body axis. Hum. Mol. Genet. 20, 1097–1110. 10.1093/hmg/ddq554 21224256

[B143] Louey S. Cock M. L. Stevenson K. M. Harding R. (2000). Placental insufficiency and fetal growth restriction lead to postnatal hypotension and altered postnatal growth in sheep. Pediatr. Res. 48, 808–814. 10.1203/00006450-200012000-00018 11102551

[B144] Louey S. Jonker S. S. Giraud G. D. Thornburg K. L. (2007). Placental insufficiency decreases cell cycle activity and terminal maturation in fetal sheep cardiomyocytes. J. Physiol. 580, 639–648. 10.1113/jphysiol.2006.122200 17234700 PMC2075561

[B145] Lumbers E. R. Boyce A. C. Joulianos G. Kumarasamy V. Barner E. Segar J. L.. (2005). Effects of cortisol on cardiac myocytes and on the expression of cardiac genes in fetal sheep. Am. J. Physiol. Regul. Integr. Comp. Physiol. 288, R567–R574. 10.1152/ajpregu.00556.2004 15576665

[B146] Lumbers E. R. Kim M. Y. Burrell J. H. Kumarasamy V. Boyce A. C. Gibson K. J.. (2009). Effects of intrafetal IGF-I on growth of cardiac myocytes in late-gestation fetal sheep. Am. J. Physiol. Endocrinol. Metab. 296, E513–E519. 10.1152/ajpendo.90497.2008 19126787

[B147] Martyn C. N. Barker D. J. Osmond C. (1996). Mothers' pelvic size, fetal growth, and death from stroke and coronary heart disease in men in the UK. Lancet 348, 1264–1268. 10.1016/S0140-6736(96)04257-2 8909378

[B148] Maruyama E. O. Lin H. Chiu S. Y. Yu H. M. Porter G. A. Hsu W. (2016). Extraembryonic but not embryonic SUMO-specific protease 2 is required for heart development. Sci. Rep. 6:20999 10.1038/srep20999 26883797 PMC4756675

[B149] Mascrez B. Ghyselinck N. B. Chambon P. Mark M. (2009). A transcriptionally silent RXRalpha supports early embryonic morphogenesis and heart development. Proc. Natl. Acad. Sci. U.S.A. 106, 4272–4277. 10.1073/pnas.0813143106 19255444 PMC2657455

[B150] Mayeur S. Silhol M. Moitrot E. Barbaux S. Breton C. Gabory A.. (2010). Placental BDNF/TrkB signaling system is modulated by fetal growth disturbances in rat and human. Placenta 31, 785–791. 10.1016/j.placenta.2010.06.008 20615547

[B151] Mayeur S. Lancel S. Theys N. Lukaszewski M. A. Duban-Deweer S. Bastide B.. (2013). Maternal calorie restriction modulates placental mitochondrial biogenesis and bioenergetic efficiency: putative involvement in fetoplacental growth defects in rats. Am. J. Physiol. Endocrinol. Metab. 304, E14–E22. 10.1152/ajpendo.00332.2012 23092912

[B152] Mayeur S. Wattez J. S. Lukaszewski M. A. Lecoutre S. Butruille L. Drougard A.. (2016). Apelin controls fetal and neonatal glucose homeostasis and is altered by maternal undernutrition. Diabetes 65, 554–560. 10.2337/db15-0228 26631739

[B153] Mess A. Zaki N. Kadyrov M. Korr H. Kaufmann P. (2007). Caviomorph placentation as a model for trophoblast invasion. Placenta 28, 1234–1238. 10.1016/j.placenta.2007.08.003 17915313

[B154] Mess A. (2007). The Guinea pig placenta: model of placental growth dynamics. Placenta 28, 812–815. 10.1016/j.placenta.2007.02.005 17382996

[B155] Miller S. L. Loose J. M. Jenkin G. Wallace E. M. (2009a). The effects of sildenafil citrate (Viagra) on uterine blood flow and well being in the intrauterine growth-restricted fetus. Am. J. Obstet. Gynecol. 200, 102.e1–102.e7. 10.1016/j.ajog.2008.08.029 18845296

[B156] Miller S. L. Supramaniam V. G. Jenkin G. Walker D. W. Wallace E. M. (2009b). Cardiovascular responses to maternal betamethasone administration in the intrauterine growth-restricted ovine fetus. Am. J. Obstet. Gynecol. 201, 613.e1–613.e8. 10.1016/j.ajog.2009.07.028 19766978

[B157] Monson T. Wright T. Galan H. L. Reynolds P. R. Arroyo J. A. (2017). Caspase dependent and independent mechanisms of apoptosis across gestation in a sheep model of placental insufficiency and intrauterine growth restriction. Apoptosis 22, 710–718. 10.1007/s10495-017-1343-9 28083721

[B158] Moreau J. L. Artap S. T. Shi H. Chapman G. Leone G. Sparrow D. B.. (2014). Cited2 is required in trophoblasts for correct placental capillary patterning. Dev. Biol. 392, 62–79. 10.1016/j.ydbio.2014.04.023 24803182

[B159] Mori A. Iwashita M. Takeda Y. (1993). Haemodynamic changes in IUGR fetus with chronic hypoxia evaluated by fetal heart-rate monitoring and Doppler measurement of blood flow velocity. Med. Biol. Eng. Comput. 31, S49–S58. 10.1007/BF02446650 8231326

[B160] Morrison J. L. Botting K. J. Dyer J. L. Williams S. J. Thornburg K. L. McMillen I. C. (2007). Restriction of placental function alters heart development in the sheep fetus. Am. J. Physiol. Regul. Integr. Comp. Physiol. 293, R306–R313. 10.1152/ajpregu.00798.2006 17428893

[B161] Morrison J. L. (2008). Sheep models of intrauterine growth restriction: fetal adaptations and consequences. Clin. Exp. Pharmacol. Physiol. 35, 730–743. 10.1111/j.1440-1681.2008.04975.x 18498533

[B162] Morton J. S. Rueda-Clausen C. F. Davidge S. T. (2010). Mechanisms of endothelium-dependent vasodilation in male and female, young and aged offspring born growth restricted. Am. J. Physiol. Regul. Integr. Comp. Physiol. 298, R930–R938. 10.1152/ajpregu.00641.2009 20053962

[B163] Mudgett J. S. Ding J. Guh-Siesel L. Chartrain N. A. Yang L. Gopal S.. (2000). Essential role for p38alpha mitogen-activated protein kinase in placental angiogenesis. Proc. Natl. Acad. Sci. U.S.A. 97, 10454–10459. 10.1073/pnas.180316397 10973481 PMC27045

[B164] Murotsuki J. Challis J. R. Han V. K. Fraher L. J. Gagnon R. (1997). Chronic fetal placental embolization and hypoxemia cause hypertension and myocardial hypertrophy in fetal sheep. Am. J. Physiol. 272, R201–R207. 10.1152/ajpregu.1997.272.1.R201 9039010

[B165] Musha Y. Itoh S. Hanson M. A. Kinoshita K. (2006). Does estrogen affect the development of abnormal vascular function in offspring of rats fed a low-protein diet in pregnancy? Pediatr. Res. 59, 784–789. 10.1203/01.pdr.0000219126.78372.c8 16641213

[B166] Myatt L. (2006). Placental adaptive responses and fetal programming. J. Physiol. 572, 25–30. 10.1113/jphysiol.2006.104968 16469781 PMC1779654

[B167] Nascimento L. Freitas C. M. Silva-Filho R. Leite A. C. Silva A. B. da Silva A. I.. (2014). The effect of maternal low-protein diet on the heart of adult offspring: role of mitochondria and oxidative stress. Appl. Physiol. Nutr. Metab. 39, 880–887. 10.1139/apnm-2013-0452 24905448

[B168] Nevin C. L. Formosa E. Maki Y. Matushewski B. Regnault T. R. H. Richardson B. S. (2018). Maternal nutrient restriction in guinea pigs as an animal model for studying growth restricted offspring with post-natal catch-up growth. Am. J. Physiol. Regul. Integr. Comp. Physiol. 314, R647–R654. 10.1152/ajpregu.00317.2017 29351419

[B169] Oh W. Omori K. Hobel C. J. Erenberg A. Emmanouilides G. C. (1975). Umbilical blood flow and glucose uptake in lamb fetus following single umbilical artery ligation. Biol. Neonate 26, 291–299. 10.1159/000240741 1169079

[B170] O'Tierney P. F. Anderson D. F. Faber J. J. Louey S. Thornburg K. L. Giraud G. D. (2010). Reduced systolic pressure load decreases cell-cycle activity in the fetal sheep heart. Am. J. Physiol. Regul. Integr. Comp. Physiol. 299, R573–R578. 10.1152/ajpregu.00754.2009 20484695 PMC2928611

[B171] Ouseph M. M. Li J. Chen H. Z. Pecot T. Wenzel P. Thompson J. C.. (2012). Atypical E2F repressors and activators coordinate placental development. Dev. Cell 22, 849–862. 10.1016/j.devcel.2012.01.013 22516201 PMC3483796

[B172] Owens J. A. Falconer J. Robinson J. S. (1987). Effect of restriction of placental growth on fetal and utero-placental metabolism. J. Dev. Physiol. 9, 225–238. 3611639

[B173] Owens J. A. Falconer J. Robinson J. S. (1989). Glucose metabolism in pregnant sheep when placental growth is restricted. Am. J. Physiol. 257, R350–R357. 10.1152/ajpregu.1989.257.2.R350 2669531

[B174] Oyama K. Padbury J. Chappell B. Martinez A. Stein H. Humme J. (1992). Single umbilical artery ligation-induced fetal growth retardation: effect on postnatal adaptation. Am. J. Physiol. 263, E575–E583. 10.1152/ajpendo.1992.263.3.E575 1415539

[B175] Paradis A. Xiao D. Zhou J. Zhang L. (2014). Endothelin-1 promotes cardiomyocyte terminal differentiation in the developing heart via heightened DNA methylation. Int. J. Med. Sci. 11, 373–380. 10.7150/ijms.7802 24578615 PMC3936032

[B176] Paradis A. N. Gay M. S. Wilson C. G. Zhang L. (2015). Newborn hypoxia/anoxia inhibits cardiomyocyte proliferation and decreases cardiomyocyte endowment in the developing heart: role of endothelin-1. PLoS ONE 10:e0116600. 10.1371/journal.pone.0116600 25692855 PMC4334650

[B177] Paulino-Silva K. M. Costa-Silva J. H. (2016). Hypertension in rat offspring subjected to perinatal protein malnutrition is not related to the baroreflex dysfunction. Clin. Exp. Pharmacol. Physiol. 43, 1046–1053. 10.1111/1440-1681.12628 27463388

[B178] Phillips I. D. Simonetta G. Owens J. A. Robinson J. S. Clarke I. J. McMillen I. C. (1996). Placental restriction alters the functional development of the pituitary-adrenal axis in the sheep fetus during late gestation. Pediatr. Res. 40, 861–866. 10.1203/00006450-199612000-00014 8947963

[B179] Phillips I. D. Anthony R. V. Simonetta G. Owens J. A. Robinson J. S. McMillen I. C. (2001). Restriction of fetal growth has a differential impact on fetal prolactin and prolactin receptor mRNA expression. J. Neuroendocrinol. 13, 175–181. 10.1046/j.1365-2826.2001.00608.x 11168843

[B180] Phillips T. J. Scott H. Menassa D. A. Bignell A. L. Sood A. Morton J. S.. (2017). Treating the placenta to prevent adverse effects of gestational hypoxia on fetal brain development. Sci. Rep. 7:9079. 10.1038/s41598-017-06300-1 28831049 PMC5567270

[B181] Poudel R. McMillen I. C. Dunn S. L. Zhang S. Morrison J. L. (2015). Impact of chronic hypoxemia on blood flow to the brain, heart, and adrenal gland in the late-gestation IUGR sheep fetus. Am. J. Physiol. Regul. Integr. Comp. Physiol. 308, R151–R162. 10.1152/ajpregu.00036.2014 25427766

[B182] Raffel G. D. Chu G. C. Jesneck J. L. Cullen D. E. Bronson R. T. Bernard O. A.. (2009). Ott1 (Rbm15) is essential for placental vascular branching morphogenesis and embryonic development of the heart and spleen. Mol. Cell. Biol. 29, 333–341. 10.1128/MCB.00370-08 18981216 PMC2612519

[B183] Rebelato H. J. Esquisatto M. A. Moraes C. Amaral M. E. Catisti R. (2013). Gestational protein restriction induces alterations in placental morphology and mitochondrial function in rats during late pregnancy. J. Mol. Histol. 44, 629–637. 10.1007/s10735-013-9522-7 23884563

[B184] Rebelato H. J. Esquisatto M. A. de Sousa Righi E. F. Catisti R. (2016). Gestational protein restriction alters cell proliferation in rat placenta. J. Mol. Histol. 47, 203–211. 10.1007/s10735-016-9660-9 26779652

[B185] Regan C. P. Li W. Boucher D. M. Spatz S. Su M. S. Kuida K. (2002). Erk5 null mice display multiple extraembryonic vascular and embryonic cardiovascular defects. Proc. Natl. Acad. Sci. U.S.A. 99, 9248–9253. 10.1073/pnas.142293999 12093914 PMC123126

[B186] Regnault T. R. Orbus R. J. Battaglia F. C. Wilkening R. B. Anthony R. V. (1999). Altered arterial concentrations of placental hormones during maximal placental growth in a model of placental insufficiency. J. Endocrinol. 162, 433–442. 10.1677/joe.0.1620433 10467235

[B187] Regnault T. R. de Vrijer B. Galan H. L. Davidsen M. L. Trembler K. A. Battaglia F. C.. (2003). The relationship between transplacental O_2_ diffusion and placental expression of PlGF, VEGF and their receptors in a placental insufficiency model of fetal growth restriction. J. Physiol. 550, 641–656. 10.1113/jphysiol.2003.039511 12740423 PMC2343042

[B188] Regnault T. R. de Vrijer B. Galan H. L. Wilkening R. B. Battaglia F. C. Meschia G. (2007). Development and mechanisms of fetal hypoxia in severe fetal growth restriction. Placenta 28, 714–723. 10.1016/j.placenta.2006.06.007 16962658

[B189] Regnault T. R. de Vrijer B. Galan H. L. Wilkening R. B. Battaglia F. C. Meschia G. (2013). Umbilical uptakes and transplacental concentration ratios of amino acids in severe fetal growth restriction. Pediatr. Res. 73, 602–611. 10.1038/pr.2013.30 23407119 PMC12254915

[B190] Rennie M. Y. Sled J. G. Adamson S. L. (2014). Effects of genes and environment on the fetoplacental arterial microcirculation in mice revealed by micro-computed tomography imaging. Microcirculation 21, 48–57. 10.1111/micc.12073 23799968

[B191] Reynolds C. M. Vickers M. H. Harrison C. J. Segovia S. A. Gray C. (2014). High fat and/or high salt intake during pregnancy alters maternal meta-inflammation and offspring growth and metabolic profiles. Physiol. Rep. 2:e12110. 10.14814/phy2.12110 25096554 PMC4246600

[B192] Reynolds C. M. Vickers M. H. Harrison C. J. Segovia S. A. Gray C. (2015). Maternal high fat and/or salt consumption induces sex-specific inflammatory and nutrient transport in the rat placenta. Physiol. Rep. 3:e12399 10.14814/phy2.12399 25991721 PMC4463828

[B193] Richter H. G. Camm E. J. Modi B. N. Naeem F. Cross C. M. Cindrova-Davies T.. (2012). Ascorbate prevents placental oxidative stress and enhances birth weight in hypoxic pregnancy in rats. J. Physiol. 590, 1377–1387. 10.1113/jphysiol.2011.226340 22289909 PMC3382329

[B194] Riley P. Anson-Cartwright L. Cross J. C. (1998). The Hand1 bHLH transcription factor is essential for placentation and cardiac morphogenesis. Nat. Genet. 18, 271–275. 10.1038/ng0398-271 9500551

[B195] Riviere G. Michaud A. Breton C. VanCamp G. Laborie C. Enache M.. (2005). Angiotensin-converting enzyme 2 (ACE2) and ACE activities display tissue-specific sensitivity to undernutrition-programmed hypertension in the adult rat. Hypertension 46, 1169–1174. 10.1161/01.HYP.0000185148.27901.fe 16203874

[B196] Roberts C. T. Sohlstrom A. Kind K. L. Earl R. A. Khong T. Y. Robinson J. S.. (2001a). Maternal food restriction reduces the exchange surface area and increases the barrier thickness of the placenta in the guinea-pig. Placenta 22, 177–185. 10.1053/plac.2000.0602 11170822

[B197] Roberts C. T. Sohlstrom A. Kind K. L. Grant P. A. Earl R. A. Robinson J. S.. (2001b). Altered placental structure induced by maternal food restriction in guinea pigs: a role for circulating IGF-II and IGFBP-2 in the mother? Placenta 22(Suppl. A), S77–S82. 10.1053/plac.2001.0643 11312635

[B198] Roberts C. T. Kind K. L. Earl R. A. Grant P. A. Robinson J. S. Sohlstrom A.. (2002). Circulating insulin-like growth factor (IGF)-I and IGF binding proteins−1 and−3 and placental development in the guinea-pig. Placenta 23, 763–770. 10.1053/plac.2002.0849 12398816

[B199] Robinson J. S. Kingston E. J. Jones C. T. Thorburn G. D. (1979). Studies on experimental growth retardation in sheep. The effect of removal of a endometrial caruncles on fetal size and metabolism. J. Dev. Physiol. 1, 379–398. 45373

[B200] Roseboom T. J. de Rooij S. R. Painter R. C. (2006). The Dutch famine and its long-tem consequences for adult health. Early Hum. Dev. 82, 485–491. 10.1016/j.earlhumdev.2006.07.001 16876341

[B201] Rossini K. F. Oliveira C. A. Rebelato H. J. Esquisatto M. A. M. Catisti R. (2017). Gestational protein restriction increases cardiac connexin 43 mRNA levels in male adult rat offspring. Arq. Bras. Cardiol. 109, 63–70. 10.5935/abc.20170081 28678925 PMC5524477

[B202] Rudolph A. M. Roman C. Gournay V. (1999). Perinatal myocardial DNA and protein changes in the lamb: effect of cortisol in the fetus. Pediatr. Res. 46, 141–146. 10.1203/00006450-199908000-00002 10447105

[B203] Rueda-Clausen C. F. Morton J. S. Davidge S. T. (2009). Effects of hypoxia-induced intrauterine growth restriction on cardiopulmonary structure and function during adulthood. Cardiovasc. Res. 81, 713–722. 10.1093/cvr/cvn341 19088083

[B204] Rueda-Clausen C. F. Morton J. S. Lopaschuk G. D. Davidge S. T. (2011). Long-term effects of intrauterine growth restriction on cardiac metabolism and susceptibility to ischaemia/reperfusion. Cardiovasc. Res. 90, 285–294. 10.1093/cvr/cvq363 21097804

[B205] Rueda-Clausen C. F. Morton J. S. Oudit G. Y. Kassiri Z. Jiang Y. Davidge S. T. (2012). Effects of hypoxia-induced intrauterine growth restriction on cardiac siderosis and oxidative stress. J. Dev. Orig. Health Dis. 3, 350–357. 10.1017/S2040174412000219 25102264

[B206] Rutland C. S. Latunde-Dada A. O. Thorpe A. Plant R. Langley-Evans S. Leach L. (2007). Effect of gestational nutrition on vascular integrity in the murine placenta. Placenta 28, 734–742. 10.1016/j.placenta.2006.07.001 16930688

[B207] Samuel J. L. Swynghedauw B. (2008). Is cardiac hypertrophy a required compemsatory mechanism in pressure-pverloaded heart? J Hypertension 26, 857–858. 10.1097/HJH.0b013e3282fbf619 18398325

[B208] Sapin V. Ward S. J. Bronner S. Chambon P. Dolle P. (1997). Differential expression of transcripts encoding retinoid binding proteins and retinoic acid receptors during placentation of the mouse. Dev. Dyn. 208, 199–210. 10.1002/(SICI)1097-0177(199702)208:2<199::AID-AJA7>3.0.CO;2-D 9022057

[B209] Sathishkumar K. Elkins R. Yallampalli U. Yallampalli C. (2009). Protein restriction during pregnancy induces hypertension and impairs endothelium-dependent vascular function in adult female offspring. J. Vasc. Res. 46, 229–239. 10.1159/000166390 18957856 PMC2860528

[B210] Sathishkumar K. Elkins R. Yallampalli U. Yallampalli C. (2012). Protein restriction during pregnancy induces hypertension in adult female rat offspring–influence of oestradiol. Br. J. Nutr. 107, 665–673. 10.1017/S0007114511003448 21787449 PMC3272136

[B211] Sathishkumar K. Balakrishnan M. P. Yallampalli C. (2015). Enhanced mesenteric arterial responsiveness to angiotensin II is androgen receptor-dependent in prenatally protein-restricted adult female rat offspring. Biol. Reprod 92:55. 10.1095/biolreprod.114.126482 25550341 PMC4342791

[B212] Scheffen I. Kaufmann P. Philippens L. Leiser R. Geisen C. Mottaghy K. (1990). Alterations of the fetal capillary bed in the guinea pig placenta following long-term hypoxia. Adv. Exp. Med. Biol. 277, 779–790. 10.1007/978-1-4684-8181-5_89 2096678

[B213] Schorpp-Kistner M. Wang Z. Q. Angel P. Wagner E. F. (1999). JunB is essential for mammalian placentation. EMBO J. 18, 934–948. 10.1093/emboj/18.4.934 10022836 PMC1171186

[B214] Schreiber M. Wang Z. Q. Jochum W. Fetka I. Elliott C. Wagner E. F. (2000). Placental vascularisation requires the AP-1 component fra1. Development 127, 4937–4948. 11044407 10.1242/dev.127.22.4937

[B215] Scotti M. Kmita M. (2012). Recruitment of 5' Hoxa genes in the allantois is essential for proper extra-embryonic function in placental mammals. Development 139, 731–739. 10.1242/dev.075408 22219351 PMC4508127

[B216] Segar J. L. Scholz T. D. Bedell K. A. Smith O. M. Huss D. J. Guillery E. N. (1997). Angiotensin AT1 receptor blockade fails to attenuate pressure-overload cardiac hypertrophy in fetal sheep. Am. J. Physiol. 273, R1501–R1508. 10.1152/ajpregu.1997.273.4.R1501 9362317

[B217] Sferruzzi-Perri A. N. Camm E. J. (2016). The programming power of the placenta. Front. Physiol. 7:33. 10.3389/fphys.2016.00033 27014074 PMC4789467

[B218] Sferruzzi-Perri A. N. Vaughan O. R. Haro M. Cooper W. N. Musial B. Charalambous M.. (2013). An obesogenic diet during mouse pregnancy modifies maternal nutrient partitioning and the fetal growth trajectory. FASEB 27, 3928–3937. 10.1096/fj.13-234823 23825226

[B219] Shah A. Matsumura N. Quon A. Morton J. S. Dyck J. R. B. Davidge S. T. (2017). Cardiovascular susceptibility to *in vivo* ischemic myocardial injury in male and female rat offspring exposed to prenatal hypoxia. Clin. Sci. 131, 2303–2317. 10.1042/CS20171122 28798077

[B220] Shaut C. A. E. Keene D. R. Sorensen L. K. Li D. Y. Stadler H. S. (2008). HOXA13 is essential for placental vascular patterning and labyrinth endothelial specification. PLoS Genet. 4:e1000073. 10.1371/journal.pgen.1000073 18483557 PMC2367452

[B221] Sherman R. C. Langley-Evans S. C. (2000). Antihypertensive treatment in early postnatal life modulates prenatal dietary influences upon blood pressure in the rat. Clin. Sci. 98, 269–275. 10.1042/cs0980269 10677384

[B222] Simonetta G. Rourke A. K. Owens J. A. Robinson J. S. McMillen I. C. (1997). Impact of placental restriction on the development of the sympathoadrenal system. Pediatr. Res. 42, 805–811. 10.1203/00006450-199712000-00015 9396562

[B223] Slater-Jefferies J. L. Lillycrop K. A. Townsend P. A. Torrens C. Hoile S. P. Hanson M. A.. (2011). Feeding a protein-restricted diet during pregnancy induces altered epigenetic regulation of peroxisomal proliferator-activated receptor-alpha in the heart of the offspring. J. Dev. Origins Adult Dis. 2, 250–255. 10.1017/S2040174410000425 22003431 PMC3191520

[B224] Snoeck A. Remacle C. Reusens B. Hoet J. J. (1990). Effect of a low protein diet during pregnancy on the fetal rat endocrine pancreas. Biol. Neonate 57, 107–118. 10.1159/000243170 2178691

[B225] Sohlstrom A. Katsman A. Kind K. L. Roberts C. T. Owens P. C. Robinson J. S.. (1998). Food restriction alters pregnancy-associated changes in IGF and IGFBP in the guinea pig. Am. J. Physiol. 274, E410–E416. 10.1152/ajpendo.1998.274.3.E410 9530122

[B226] Soonpaa M. H. Kim K. K. Pajak L. Franklin M. Field L. J. (1996). Cardiomyocyte DNA synthesis and binucleation during murine development. Am. J. Physiol. 271, H2183–H2189. 10.1152/ajpheart.1996.271.5.H2183 8945939

[B227] Strakovsky R. S. Zhou D. Pan Y. X. (2010). A low-protein diet during gestation in rats activates the placental mammalian amino acid response pathway and programs the growth capacity of offspring. J. Nutr. 140, 2116–2120. 10.3945/jn.110.127803 20980649

[B228] Stumpo D. J. Byrd N. A. Phillips R. S. Ghosh S. Maronpot R. R. Castranio T.. (2004). Chorioallantoic fusion defects and embryonic lethality resulting from disruption of Zfp36L1, a gene encoding a CCCH tandem zinc finger protein of the Tristetraprolin family. Mol. Cell. Biol. 24, 6445–6455. 10.1128/MCB.24.14.6445-6455.2004 15226444 PMC434251

[B229] Sundgren N. C. Giraud G. D. Schultz J. M. Lasarev M. R. Stork P. S. Thornburg K. L. (2003). Extracellular signal-regulated kinase and phosphoinositol-3 kinase mediate IGF-1 induced proliferation of fetal sheep cardiomyocytes. Am. J. Physiol. Regul. Integr. Comp. Physiol. 285, R1481–R1489. 10.1152/ajpregu.00232.2003 12947030

[B230] Supramaniam V. G. Jenkin G. Loose J. Wallace E. M. Miller S. L. (2006). Chronic fetal hypoxia increases activin A concentrations in the late-pregnant sheep. Br. J. Obstet. Gynaecol. 113, 102–109. 10.1111/j.1471-0528.2005.00791.x 16398778

[B231] Szabova L. Son M. Y. Shi J. Sramko M. Yamada S. S. Swaim W. D.. (2010). Membrane-type MMPs are indispensable for placental labyrinth formation and development. Blood 116, 5752–5761. 10.1182/blood-2009-10-249847 20858856 PMC3031418

[B232] Tai-Nagara I. Yoshikawa Y. Numata N. Ando T. Okabe K. Sugiura Y.. (2017). Placental labyrinth formation in mice requires endothelial FLRT2/UNC5B signaling. Development 144, 2392–2401. 10.1242/dev.149757 28576770

[B233] Takeda K. Ho V. C. Takeda H. Duan L. J. Nagy A. Fong G. H. (2006). Placental but not heart defects are associated with elevated hypoxia-inducible factor alpha levels in mice lacking prolyl hydroxylase domain protein 2. Mol. Cell. Biol. 26, 8336–8346. 10.1128/MCB.00425-06 16966370 PMC1636770

[B234] Tanaka H. Nagaike K. Takeda N. Itoh H. Kohama K. Fukushima T.. (2005). Hepatocyte growth factor activator inhibitor type 1 (HAI-1) is required for branching morphogenesis in the chorioallantoic placenta. Mol. Cell. Biol. 25, 5687–5698. 10.1128/MCB.25.13.5687-5698.2005 15964823 PMC1157006

[B235] Tare M. Parkington H. C. Wallace E. M. Sutherland A. E. Lim R. Yawno T.. (2014). Maternal melatonin administration mitigates coronary stiffness and endothelial dysfunction, and improves heart resilience to insult in growth restricted lambs. J. Physiol. 592, 2695–2709. 10.1113/jphysiol.2014.270934 24710061 PMC4080947

[B236] Thompson L. P. Dong Y. (2005). Chronic hypoxia decreases endothelial nitric oxide synthase protein expression in fetal guinea pig hearts. J. Soc. Gynecol. Investig. 12, 388–395. 10.1016/j.jsgi.2005.04.011 15982907

[B237] Thompson R. S. Trudinger B. J. (1990). Doppler waveform pulsatility index and resistance, pressure and flow in the umbilical placental circulation: an investigation using a mathematical model. Ultrasound Med. Biol. 16, 449–458. 10.1016/0301-5629(90)90167-B 2238251

[B238] Thompson L. P. Aguan K. Zhou H. (2004). Chronic hypoxia inhibits contraction of fetal arteries by increased endothelium-derived nitric oxide and prostaglandin synthesis. J. Soc. Gynecol. Investig. 11, 511–520. 10.1016/j.jsgi.2004.05.008 15582495

[B239] Thompson L. Dong Y. Evans L. (2009). Chronic hypoxia increases inducible NOS-derived nitric oxide in fetal guinea pig hearts. Pediatr. Res. 65, 188–192. 10.1203/PDR.0b013e31818d6ad0 19047955 PMC6314287

[B240] Thompson J. A. Richardson B. S. Gagnon R. Regnault T. R. (2011). Chronic intrauterine hypoxia interferes with aortic development in the late gestation ovine fetus. J. Physiol. 589, 3319–3332. 10.1113/jphysiol.2011.210625 21540340 PMC3145942

[B241] Thompson J. A. Piorkowska K. Gagnon R. Richardson B. S. Regnault T. R. (2013). Increased collagen deposition in the heart of chronically hypoxic ovine fetuses. J. Dev. Orig. Health Dis. 4, 470–478. 10.1017/S2040174413000299 24924226

[B242] Thompson L. P. Pence L. Pinkas G. Song H. Telugu B. P. (2016). Placental hypoxia during early pregnancy causes maternal hypertension and placental insufficiency in the hypoxic guinea pig model. Biol. Reprod. 95:128. 10.1095/biolreprod.116.142273 27806942 PMC5315426

[B243] Thureen P. J. Trembler K. A. Meschia G. Makowski E. L. Wilkening R. B. (1992). Placental glucose transport in heat-induced fetal growth retardation. Am. J. Physiol. 263, R578–R585. 10.1152/ajpregu.1992.263.3.R578 1415644

[B244] Tong W. Xue Q. Li Y. Zhang L. (2011). Maternal hypoxia alters matrix metalloproteinase expression patterns and causes cardiac remodeling in fetal and neonatal rats. Am. J. Physiol. Heart Circ. Physiol. 301, H2113–H2121. 10.1152/ajpheart.00356.2011 21856922 PMC3213965

[B245] Torrens C. Brawley L. Barker A. C. Itoh S. Poston L. Hanson M. A. (2003). Maternal protein restriction in the rat impairs resistance artery but not conduit artery function in pregnant offspring. J. Physiol. 547, 77–84. 10.1113/jphysiol.2002.026120 12562942 PMC2342611

[B246] Torrens C. Brawley L. Anthony F. W. Dance C. S. Dunn R. Jackson A. A.. (2006). Folate supplementation during pregnancy improves offspring cardiovascular dysfunction induced by protein restriction. Hypertension 47, 982–987. 10.1161/01.HYP.0000215580.43711.d1 16585422

[B247] Torrens C. Poston L. Hanson M. A. (2008). Transmission of raised blood pressure and endothelial dysfunction to the F2 generation induced by maternal protein restriction in the F0, in the absence of dietary challenge in the F1 generation. Br. J. Nutr. 100, 760–766. 10.1017/S0007114508921747 18304387

[B248] Trudinger B. J. Giles W. B. Cook C. M. Bombardieri J. Collins L. (1985). Fetal umbilical artery flow velocity waveforms and placental resistance: clinical significance. Br. J. Obstet. Gynaecol. 92, 23–30. 10.1111/j.1471-0528.1985.tb01044.x 4038455

[B249] Trudinger B. J. Stevens D. Connelly A. Hales J. R. Alexander G. Bradley L.. (1987). Umbilical artery flow velocity waveforms and placental resistance: the effects of embolization of the umbilical circulation. Am. J. Obstet. Gynecol. 157, 1443–1448. 10.1016/S0002-9378(87)80241-7 2962497

[B250] Tucker D. C. (1985). Components of functional sympathetic control of heart rate in neonatal rats. Am. J. Physiol. 248, R601–R610. 10.1152/ajpregu.1985.248.5.R601 2859811

[B251] Unezaki S. Horai R. Sudo K. Iwakura Y. Ito S. (2007). Ovol2/Movo, a homologue of Drosophila ovo, is required for angiogenesis, heart formation and placental development in mice. Genes Cells 12, 773–785. 10.1111/j.1365-2443.2007.01084.x 17573777

[B252] Vickers M. H. Breier B. H. Cutfield W. S. Hofman P. L. Gluckman P. D. (2000). Fetal origins of hyperphagia, obesity, and hypertension and postnatal amplification by hypercaloric nutrition. Am. J. Physiol. Endocrinol. Metab. 279, E83–E87. 10.1152/ajpendo.2000.279.1.E83 10893326

[B253] Vickers M. H. Ikenasio B. A. Breier B. H. (2002). Adult growth hormone treatment reduces hypertension and obesity induced by an adverse prenatal environment. J. Endocrinol. 175, 615–623. 10.1677/joe.0.1750615 12475373

[B254] Vranas S. Heinemann G. K. Liu H. De Blasio M. J. Owens J. A. Gatford K. L.. (2017). Small size at birth predicts decreased cardiomyocyte number in the adult ovine heart. J. Dev. Orig. Health Dis. 8, 618–625. 10.1017/S2040174417000381 28975880

[B255] Wadley G. D. Wlodek M. E. Ng G. Goodman C. Stathis C. McConell G. K. (2010). Growth restriction before and after birth increases kinase signaling pathways in the adult rat heart. J. Dev. Orig. Health Dis. 1, 376–385. 10.1017/S2040174410000607 25142009

[B256] Wadley G. D. McConell G. K. Goodman C. A. Siebel A. L. Westcott K. T. Wlodek M. E. (2013). Growth restriction in the rat alters expression of metabolic genes during postnatal cardiac development in a sex-specific manner. Physiol. Genomics 45, 99–105. 10.1152/physiolgenomics.00095.2012 23232075

[B257] Wadley G. D. Laker R. C. McConell G. K. Wlodek M. E. (2016). Endurance training in early life results in long-term programming of heart mass in rats. Physiol. Rep. 4:e12720. 10.14814/phy2.12720 26893473 PMC4759045

[B258] Wakefield S. L. Lane M. Mitchell M. (2011). Impaired mitochondrial function in the preimplantation embryo perturbs fetal and placental development in the mouse. Biol. Reprod. 84, 572–580. 10.1095/biolreprod.110.087262 21076083

[B259] Walker D. W. Davies A. N. McMillen I. C. (1990). Effect of hyperthermia on the plasma concentrations of prolactin and cortisol in the fetal lamb and pregnant ewe during late gestation. J. Dev. Physiol. 13, 173–177. 2277182

[B260] Walker B. R. (2007). Glucocorticoids and cardiovascular disease. Eur. J. Endocrinol. 157, 545–559. 10.1530/EJE-07-0455 17984234

[B261] Wang K. C. Zhang L. McMillen I. C. Botting K. J. Duffield J. A. Zhang S.. (2011). Fetal growth restriction and the programming of heart growth and cardiac insulin-like growth factor 2 expression in the lamb. J. Physiol. 589, 4709–4722. 10.1113/jphysiol.2011.211185 21807611 PMC3213418

[B262] Wang K. C. Brooks D. A. Botting K. J. Morrison J. L. (2012). IGF-2R-mediated signaling results in hypertrophy of cultured cardiomyocytes from fetal sheep. Biol. Reprod. 86:183 10.1095/biolreprod.112.100388 22441800

[B263] Wang K. C. Lim C. H. McMillen I. C. Duffield J. A. Brooks D. A. Morrison J. L. (2013). Alteration of cardiac glucose metabolism in association to low birth weight: experimental evidence in lambs with left ventricular hypertrophy. Metab. Clin. Exp. 62, 1662–1672. 10.1016/j.metabol.2013.06.013 23928106

[B264] Wang K. C. Brooks D. A. Summers-Pearce B. Bobrovskaya L. Tosh D. N. Duffield J. A.. (2015a). Low birth weight activates the renin-angiotensin system, but limits cardiac angiogenesis in early postnatal life. Physiol. Rep. 3:e12270. 10.14814/phy2.12270 25649246 PMC4393187

[B265] Wang K. C. Tosh D. N. Zhang S. McMillen I. C. Duffield J. A. Brooks D. A.. (2015b). IGF-2R-Galphaq signaling and cardiac hypertrophy in the low-birth-weight lamb. Am. J. Physiol. Regul. Integr. Comp. Physiol. 308, R627–R635. 10.1152/ajpregu.00346.2014 25632020 PMC4385999

[B266] Watkins A. J. Ursell E. Panton R. Papenbrock T. Hollis L. Cunningham C.. (2008). Adaptive responses by mouse early embryos to maternal diet protect fetal growth but predispose to adult onset disease. Biol. Reprod. 78, 299–306. 10.1095/biolreprod.107.064220 17989357

[B267] Watkins A. J. Lucas E. S. Wilkins A. Cagampang F. R. Fleming T. P. (2011). Maternal periconceptional and gestational low protein diet affects mouse offspring growth, cardiovascular and adipose phenotype at 1 year of age. PloS One 6:e28745. 10.1371/journal.pone.0028745 22194901 PMC3240629

[B268] Watkins A. J. Lucas E. S. Marfy-Smith S. Bates N. Kimber S. J. Fleming T. P. (2015). Maternal nutrition modifies trophoblast giant cell phenotype and fetal growth in mice. Reproduction 149, 563–575. 10.1530/REP-14-0667 25755287 PMC4418750

[B269] Wattez J. S. Delahaye F. Barella L. F. Dickes-Coopman A. Montel V. Breton C.. (2014). Short- and long-term effects of maternal perinatal undernutrition are lowered by cross-fostering during lactation in the male rat. J. Dev. Origins Adult Dis. 5, 109–120. 10.1017/S2040174413000548 24847697

[B270] Wendling O. Chambon P. Mark M. (1999). Retinoid, X.,receptors are essential for early mouse development and placentogenesis. Proc Natl Acad Sci. U.S.A. 96, 547–551. 9892670 10.1073/pnas.96.2.547 PMC15173

[B271] Wenzel P. L. Wu L. de Bruin A. Chong J. L. Chen W. Y. Dureska G.. (2007). Rb is critical in a mammalian tissue stem cell population. Genes Dev. 21, 85–97. 10.1101/gad.1485307 17210791 PMC1759903

[B272] Wigglesworth J. S. (1974). Fetal growth retardation. Animal model: uterine vessel ligation in the pregnant rat. Am. J. Pathol. 77, 347–350. 4471602 PMC1910905

[B273] Withington S. L. Scott A. N. Saunders D. N. Lopes Floro K. Preis J. I. Michalicek J.. (2006). Loss of Cited2 affects trophoblast formation and vascularization of the mouse placenta. Dev. Biol. 294, 67–82. 10.1016/j.ydbio.2006.02.025 16579983

[B274] Wlodek M. E. Westcott K. T. O'Dowd R. Serruto A. Wassef L. Moritz K. M.. (2005). Uteroplacental restriction in the rat impairs fetal growth in association with alterations in placental growth factors including PTHrP. Am. J. Physiol. Regul. Integr. Comp. Physiol. 288, R1620–R1627. 10.1152/ajpregu.00789.2004 15661964

[B275] Woodall S. M. Breier B. H. Johnston B. M. Gluckman P. D. (1996a). A model of intrauterine growth retardation caused by chronic maternal undernutrition in the rat: effects on the somatotrophic axis and postnatal growth. J. Endocrinol. 150, 231–242. 8869590 10.1677/joe.0.1500231

[B276] Woodall S. M. Johnston B. M. Breier B. H. Gluckman P. D. (1996b). Chronic maternal undernutrition in the rat leads to delayed postnatal growth and elevated blood pressure of offspring. Pediatr. Res. 40, 438–443. 8865281 10.1203/00006450-199609000-00012

[B277] Woodall S. M. Breier B. H. Johnston B. M. Bassett N. S. Barnard R. Gluckman P. D. (1999). Administration of growth hormone or IGF-I to pregnant rats on a reduced diet throughout pregnancy does not prevent fetal intrauterine growth retardation and elevated blood pressure in adult offspring. J. Endocrinol. 163, 69–77. 10.1677/joe.0.1630069 10495409

[B278] Wooding F. B. P. Burton G. J. (2008). Comparative Placentation: Structures, Functions, and Evolution, 1Edn. Berlin; Heidelberg: Springer-Verlag.

[B279] Wu L. de Bruin A. Saavedra H. I. Starovic M. Trimboli A. Yang Y.. (2003). Extra-embryonic function of Rb is essential for embryonic development and viability. Nature 421, 942–947. 10.1038/nature01417 12607001

[B280] Xiong F. Lin T. Song M. Ma Q. Martinez S. R. Lv J.. (2016). Antenatal hypoxia induces epigenetic repression of glucocorticoid receptor and promotes ischemic-sensitive phenotype in the developing heart. J. Mol. Cell. Cardiol. 91, 160–171. 10.1016/j.yjmcc.2016.01.003 26779948 PMC4764467

[B281] Xu Y. Williams S. J. O'Brien D. Davidge S. T. (2006). Hypoxia or nutrient restriction during pregnancy in rats leads to progressive cardiac remodeling and impairs postischemic recovery in adult male offspring. FASEB J. 20, 1251–1253. 10.1096/fj.05-4917fje 16632594

[B282] Xue Q. Zhang L. (2009). Prenatal hypoxia causes a sex-dependent increase in heart susceptibility to ischemia and reperfusion injury in adult male offspring: role of protein kinase C epsilon. J. Pharmacol. Exp. Ther. 330, 624–632. 10.1124/jpet.109.153239 19470841 PMC2713094

[B283] Yang J. Boerm M. McCarty M. Bucana C. Fidler I. J. Zhuang Y.. (2000). Mekk3 is essential for early embryonic cardiovascular development. Nat. Genet. 24, 309–313. 10.1038/73550 10700190

[B284] Yiallourou S. R. Witcombe N. B. Sands S. A. Walker A. M. Horne R. S. (2013). The development of autonomic cardiovascular control is altered by preterm birth. Early Hum. Dev. 89, 145–152. 10.1016/j.earlhumdev.2012.09.009 23058299

[B285] Zhang S. Barker P. Botting K. J. Roberts C. T. McMillan C. M. McMillen I. C.. (2016). Early restriction of placental growth results in placental structural and gene expression changes in late gestation independent of fetal hypoxemia. Physiol. Rep. 4:e13049. 10.14814/phy2.13049 27923976 PMC5357827

[B286] Zhou J. Xiao D. Hu Y. Wang Z. Paradis A. Mata-Greenwood E.. (2013). Gestational hypoxia induces preeclampsia-like symptoms via heightened endothelin-1 signaling in pregnant rats. Hypertension 62, 599–607. 10.1161/HYPERTENSIONAHA.113.01449 23817493 PMC3826959

[B287] Ziebell B. T. Galan H. L. Anthony R. V. Regnault T. R. Parker T. A. Arroyo J. A. (2007). Ontogeny of endothelial nitric oxide synthase mRNA in an ovine model of fetal and placental growth restriction. Am. J. Obstet. Gynecol. 197, 420.e1–420.e5. 10.1016/j.ajog.2007.07.016 17904986

